# Using Genomics To Investigate an Outbreak of Vancomycin-Resistant Enterococcus faecium ST78 at a Large Tertiary Hospital in Queensland

**DOI:** 10.1128/spectrum.04204-22

**Published:** 2023-05-16

**Authors:** Budi Permana, Patrick N. A. Harris, Naomi Runnegar, Margaret Lindsay, Belinda C. Henderson, E. G. Playford, David L. Paterson, Scott A. Beatson, Brian M. Forde

**Affiliations:** a School of Chemistry and Molecular Biosciences, Faculty of Science, The University of Queensland, Brisbane, Australia; b Australian Centre for Ecogenomics, The University of Queensland, Brisbane, Australia; c Australian Infectious Disease Research Centre, Faculty of Science, The University of Queensland, Brisbane, Australia; d University of Queensland Centre for Clinical Research, Faculty of Medicine, The University of Queensland, Brisbane, Australia; e Pathology Queensland, Central Laboratory, Brisbane, Australia; f Princess Alexandra–Southside Clinical School, Faculty of Medicine, The University of Queensland, Brisbane, Australia; g Infection Management Services, Princess Alexandra Hospital, Brisbane, Australia; h Herston Infectious Diseases Institute, Metro North Health, Brisbane, Australia; University of Arkansas for Medical Sciences

**Keywords:** vancomycin resistance, *Enterococcus faecium*, VRE, ST78, whole-genome sequencing, genomic epidemiology, surveillance, *Enterococcus*, epidemiology, genomics, outbreak, transmission

## Abstract

To investigate an outbreak of vancomycin-resistant Enterococcus faecium (VREfm) sequence type 78 (ST78) in a large tertiary Australian hospital. A collection of 63 VREfm ST78 isolates, identified during a routine genomic surveillance program, were subjected to genomic epidemiological analysis based on whole-genome sequencing (WGS) data. The population structure was reconstructed using phylogenetic analysis, and a collection of publicly available VREfm ST78 genomes were used to provide global context. Core genome single nucleotide polymorphism (SNP) distances and available clinical metadata were used to characterize outbreak clusters and reconstruct transmission events. *In silico* genotyping confirmed that all study isolates were *vanB*-type VREfm carrying virulence characteristics of the hospital-associated E. faecium. Phylogenetic analysis identified two distinct phylogenetic clades, only one of which was responsible for a hospital outbreak. Four outbreak subtypes could be defined with examples of recent transmissions. Inference on transmission trees suggested complex transmission routes with unknown environmental reservoirs mediating the outbreak. WGS-based cluster analysis with publicly available genomes identified closely related Australian ST78 and ST203 isolates, highlighting the capacity for WGS to resolve complex clonal relationships between the VREfm lineages. Whole genome-based analysis has provided a high-resolution description of an outbreak of *vanB*-type VREfm ST78 in a Queensland hospital. Combined routine genomic surveillance and epidemiological analysis have facilitated better understanding of the local epidemiology of this endemic strain, providing valuable insight for better targeted control of VREfm.

**IMPORTANCE** Vancomycin-resistant Enterococcus faecium (VREfm) is a leading cause of health care-associated infections (HAIs) globally. In Australia, the spread of hospital-adapted VREfm is largely driven by a single clonal group (clonal complex [CC]), CC17, to which the lineage ST78 belongs. While implementing a genomic surveillance program in Queensland, we observed increased incidence of ST78 colonizations and infections among patients. Here, we demonstrate the use of real-time genomic surveillance as a tool to support and enhance infection control (IC) practices. Our results show that real-time whole-genome sequencing (WGS) can efficiently disrupt outbreaks by identifying transmission routes that in turn can be targeted using resource-limited interventions. Additionally, we demonstrate that by placing local outbreaks in a global context, high-risk clones can be identified and targeted prior to them becoming established within clinical environments. Finally, the persistence of these organism within the hospital highlights the need for routine genomic surveillance as a management tool to control VRE transmission.

## INTRODUCTION

Vancomycin-resistant Enterococcus faecium (VREfm) is a frequent cause of health care-associated infections (HAIs). First reported in the United Kingdom and France in 1988 (1, 2), VREfm is rapidly emerging as a major causative agent of bloodstream infections worldwide (3, 4). The intrinsic and acquired resistance to several antibiotics particularly to aminoglycosides and glycopeptides make VREfm difficult to treat and pose a serious threat to immunocompromised patients in many hospitals (5, 6). VREfm is now classified as a global health threat and has been listed by the American Center for Disease Control (CDC) and the World Health Organization (WHO) as a high priority target for antimicrobial research and surveillance (7–9).

E. faecium clonal complex 17 (CC17) is the main cause of hospital-associated VREfm infection. VREfm CC17 is a polyclonal group consisting of multiple sequence types (STs) that share numerous traits to promote their survival and adaptation to the hospital environment (10). These include resistance to vancomycin, conferred by *van* operons, and mobile genetic elements mediating the spread of resistance through horizontal gene transfer (11, 12). They are enriched for virulence factors such as enterococcal surface protein *esp* (13), the hyaluronidase gene *hyl* (14), the collagen adhesin gene *acm* (15), and several genes encoding pili and microbial surface components recognizing adhesive matrix molecules (MSCRAMMs) (16).

In Australia, the first clinical VREfm was reported in 1994 isolated from a liver transplant recipient at Austin Hospital in Melbourne (17). Since then, multiple health care-associated VREfm outbreaks have been reported throughout Australia (18–21). In Australia, the 2015 to 2019 Enterococcal Sepsis Outcome Program (AESOP) conducted by the Australian Group on Antimicrobial Resistance (AGAR) identified a number of CC17 lineages that were prevalent in Australia, including ST17, ST1421, ST1424, ST796, ST203, ST78, ST80, and ST262 (22–25). The ST78 lineage is primarily found in Queensland and New South Wales (22–25) and was recently reported in Victoria (25).

In Queensland, through an active genomic surveillance program supported by Queensland Health (26, 27), we observed an increased number of VREfm ST78-colonized cases in a large tertiary hospital. The frequency of identification and genomic relatedness of these isolates was concordant with a developing outbreak. To better understand the epidemiological dynamics of this suspected outbreak, we investigated all 63 E. faecium ST78 genomes available in our surveillance database that were collected from the hospital over a period of 815 days. This paper described a detailed use of whole-genome sequencing (WGS) and genomic epidemiology to characterize VREfm ST78 clustering and potential transmissions within the hospital setting.

## RESULTS

### Case study.

**FIG 1:**
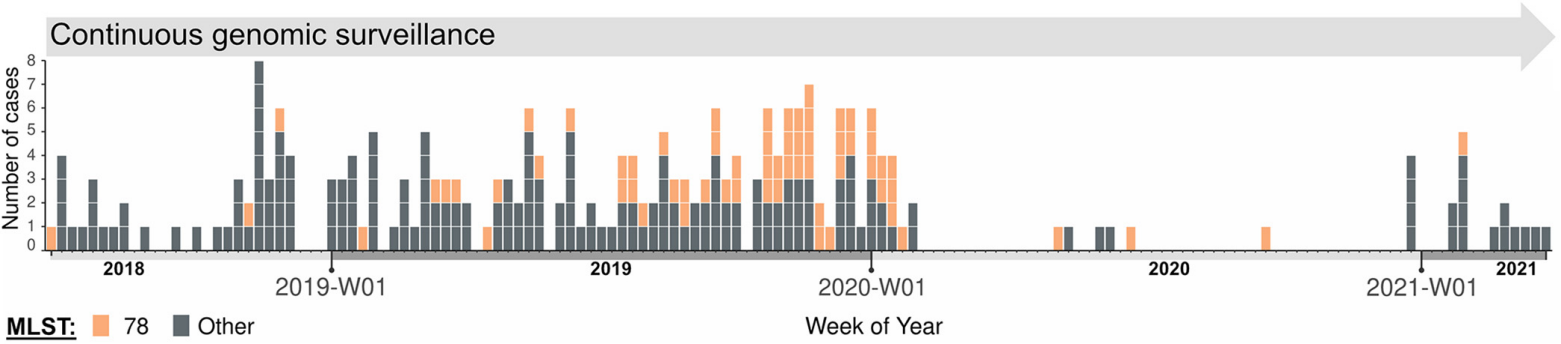
Epidemic curve of vancomycin-resistant E. faecium (VREfm) ST78 from the study hospital. The horizontal and vertical axes represent collection date (scaled to weekly) and the number of the cases, respectively. MLST, multilocus sequence typing.

Between 30 June 2018 and 21 September 2020, a total of 63 VREfm ST78 isolates were cultured from 62 patients (P) and 58 environmental (E) samples (Fig. 1; Fig. S6). The majority of samples were collected from colonized patients (62 of 63) with only a single positive environmental sample (Table S2). Patient isolates were primarily from routine surveillance (rectal) swabs (*n* = 55) with the remainder collected from both noninvasive (*n* = 5) and invasive (bloodstream infection [BSI]; *n* = 2) clinical specimens. The positive environmental isolate was identified in a toilet attached to a room occupied by a patient who was positive for carriage of VREfm ST78.

### All study isolates were *vanB*-type VREfm.

*In silico* resistance gene profiling confirmed that all study isolates carried the *vanB* gene conferring vancomycin resistance (28). The *vanB* locus is generally associated with the chromosomally located Tn*1549*-/Tn*5382*-subtype conjugative transposon (3, 29). Similar to previous observations (29, 30), the *vanB* locus was not associated with plasmid carriage among our clinical isolates (Table S5). The *vanB* locus was located on a Tn*1549*-like transposon and well conserved among Queensland ST78. Some variation was observed in the *vanB* operon between clade 1 and 2, with clade 2 isolates carrying an IS110 insertion in between *vanW* and *vanHB* genes (Fig. S7). In addition to *vanB*, all isolates carried genes conferring resistance to aminoglycosides (*aac(6′)*-Ii [31]), macrolides (*msr(C)* [32]), and tetracycline (*tet(L)* and *tet(M)* [33]) (Table S6) and several common virulence genes (supplemental materials).

### Interstate and international carriage contribute to emergence of ST78 in Queensland.

**FIG 2:**
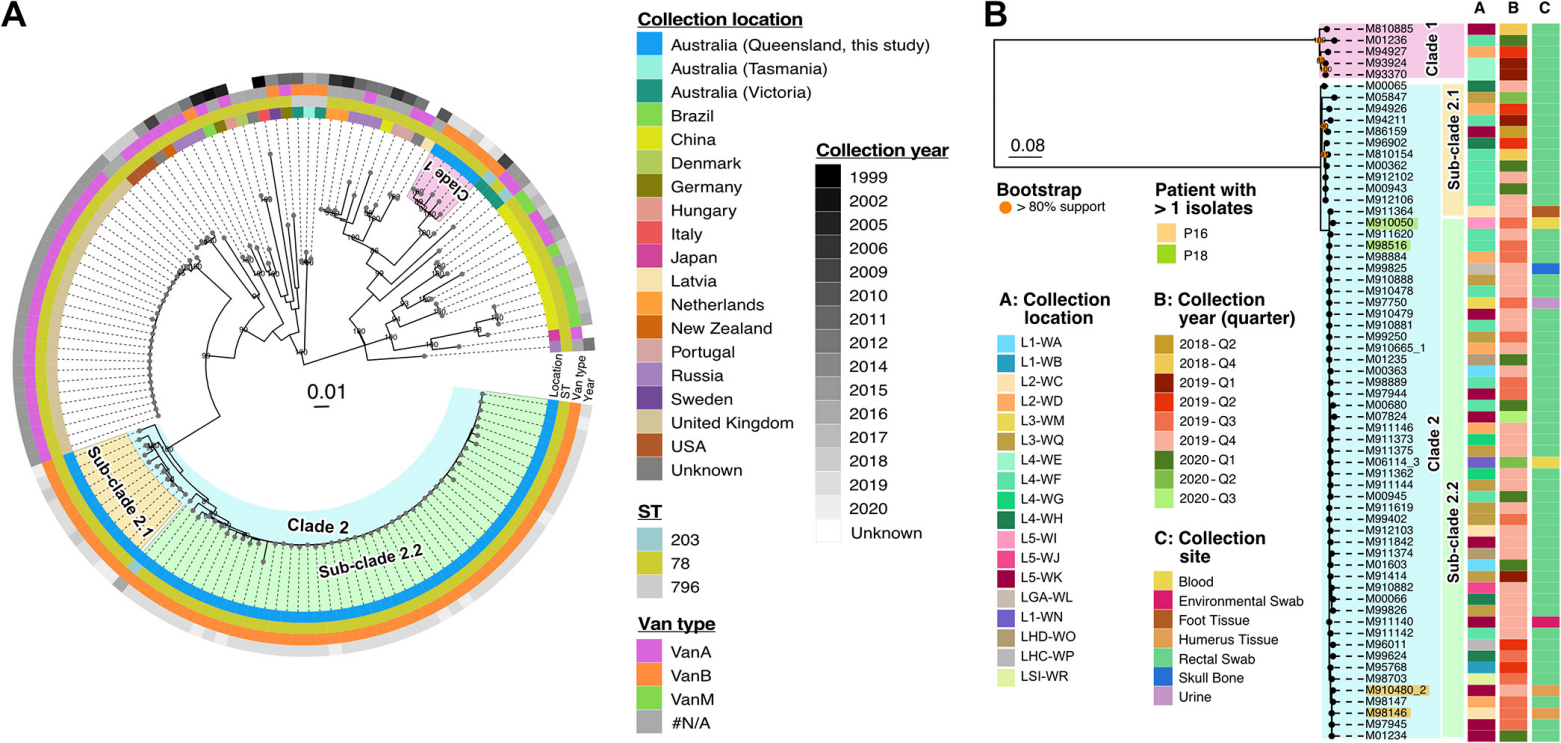
(A) Midpoint-rooted maximum likelihood phylogenetic tree of 63 VREfm study isolates and an additional 73 publicly available E. faecium genomes based on 1,732 recombination-adjusted core genome single nucleotide polymorphism (SNPs). (B) Midpoint-rooted maximum likelihood phylogenetic tree of 63 VREfm ST78 only constructed from 344 recombination-adjusted core genome SNPs. Isolate collection location (column A), year of collection (column B), and collection site (column C) are indicated by the colored rectangles. Highlighted tip label indicates isolates from the same patient (patient 16 [P16] and patient 18 [P18]; see key). The bar on the trees represents nucleotide substitutions per site.

Phylogenetic analysis of outbreak isolates in the context of global strains provides important information on the origins of the ST78 in Queensland. Queensland isolates were separated into two distinct phylogenetic clades (clade 1 and 2; Fig. 2B) separated by 253 recombination-adjusted core genome single nucleotide polymorphism (SNPs) (or 1,244 SNPs before recombination filtering; Table S7). Clade 1, the smaller of the two Queensland clades, consists of five isolates and shares a recent ancestor with an ST203 isolate (GCF_000444405.1) collected in Melbourne in 2009 (19). ST203 is the single locus variant (SLV) of ST78 and was included in this comparison to provide more regional context. Global cluster analysis from NCBI Pathogen Detection shows that ST78 clade 1 isolates were also closely clustered with other *vanB*-type ST78 isolates from Australia collected as early as 2015 (cluster ID PDS000062997).

Clade 2, to which most isolates belong (92%), broadly shared a common ancestor with a group of *vanA*-type ST78 isolates collected from a single hospital in the United Kingdom in 2015 (60) (Fig. 2A). Clade 2 can be further separated into two subclades (2.1 and 2.2) (Fig. 2A). Analysis using core genome multilocus sequence typing (cgMLST) against global clusters identified in NCBI Pathogen Detection (34) showed that clades 2.1 and 2.2 were closely related with two SNP clusters of *vanB*-type ST78 collected from Australia between 2014 to 2017 (Fig. S3). Interestingly, based on our cgMLST analysis, another ST203 isolate (GCF_003957785.1) collected at a different Brisbane hospital in 2016 (35) was found to cluster with clade 2 isolates (Fig. 2A; Fig. S3).

### Clade 2 is associated with high-level in-hospital transmission.

**FIG 3:**
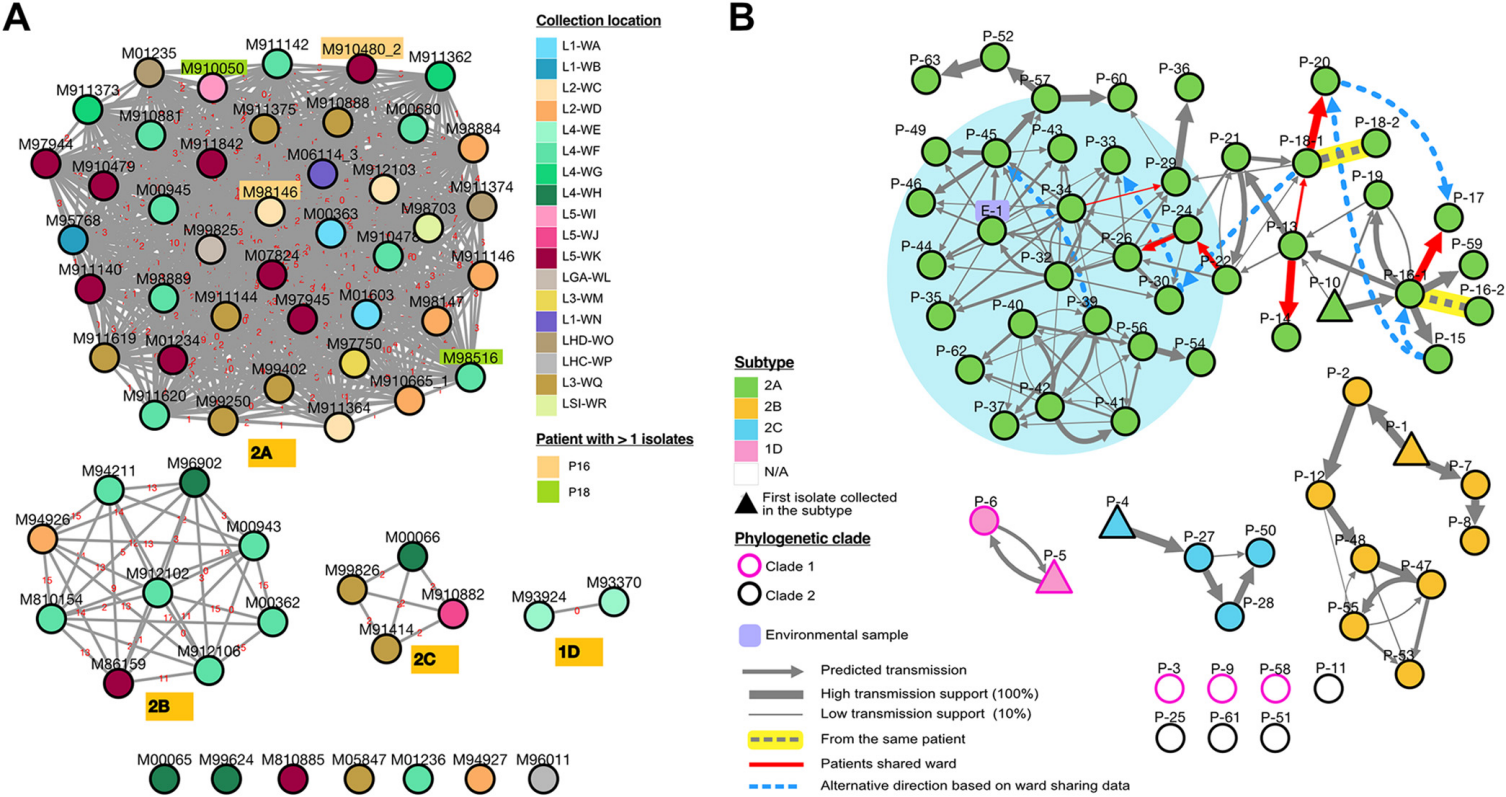
(A) Network visualization of four VREfm ST78 subtypes based on SNP distance cutoff of ≤20 SNPs. The nodes represent isolates, and the lines show the number of SNP distance displayed by the red text. The color of the node represents the location of collection (ward). A highlighted node label indicates an isolate from the same patient. An interactive version of these clusters can be viewed in GraphSNP website (https://graphsnp.fordelab.com; see supplemental methods for instructions). (B) Transmission networks based on collection dates, patient ward sharing, and pairwise SNP distance within the same subtype. The nodes represent patients, and the lines denote inferred transmission relationships. Transmission support based on Outbreaker2 model is indicated by the thickness of the line. Red lines indicate transmission between patients who shared wards, and dashed blue lines denote alternative transmission directions based on wardsharing data.

High-resolution, network-based clustering, with a previously defined SNP cutoff of ≤20 SNPs (36) was used to identify VREfm subtypes among the study isolates (see Materials and Methods for subtyping approach). Three genetically distinct subtypes were identified within clade 2 (subtypes 2A, 2B, and 2C; Fig. 3A). 2A is the largest of the clade 2 subtypes (*n* = 41). Isolates within subtype 2A were very closely related (median, 3 SNPs; interquartile range [IQR], 1 to 5 SNPs [before recombination filtering]; Table S8), suggesting recent transmission between patients (36). However, despite the strength of isolate relatedness within subtype 2A, the chain of transmission could not be reliably predicted, with most isolates forming a dense tangled network with multiple predicted routes of transmission (Fig. 3B). Although 2A isolates were closely related genetically, most were spatially dispersed throughout the hospital, indicating that in addition to patient-to-patient transmission, environmental or other unknown reservoirs have likely contributed to the spread of this subtype.

In contrast, isolates from subtype 2B (*n* = 9) were more genetically diverse (median, 12.5 SNPs; IQR, 4.5 to 15 SNPs) but largely confined to a single ward. Although some direct patient-to-patient transfer is likely, most isolates fall outside the SNP cutoff (≤6 SNPs) used to define recent transmission between patients (36). However, the observed diversity does not rule out the existence of an unsampled reservoir that may have been present in the local environment for some time.

The last and the smallest subtype from clade 2 is 2C (*n* = 4), having also a minimal genetic diversity (median, 2 SNPs; IQR, 2 SNPs). Two 2C isolates collected from different patients in an intensive care unit (ICU; L3-WQ in Fig. 2B) are closely related genetically, despite distant sampling dates (252 days), suggesting an indirect transmission events and further supporting the presence of within-hospital reservoirs.

A single recent transmission event was also observed within clade 1. Isolates M93924 and M93370 were found to have identical core genomes (0 SNPs; subtype 1D in Fig. 3A) and were collected from individuals (patient 6 [P6] and P5, respectively) admitted to the same ward, suggesting direct transmission between these individuals (Fig. 3B, pink nodes). However, patients were not admitted to the ward at the same time, again supporting the presence of an unsampled intermediary in the chain of transmission.

## DISCUSSION

Here, we present a prospective analysis using WGS and detailed patient epidemiological data to provide high-resolution insights into the origin, population structure, and transmission of VREfm ST78 within a major Queensland hospital. Our analysis identified two independent introductions of ST78 into the hospital with only one population, clade 2, becoming successfully established. The two populations were indistinguishable by MLST and *van* typing but identified as distinct lineages based on WGS data, highlighting the superiority of WGS for accurate identification of high-risk VREfm clones. Expanding the analysis to include historical regional clinical cases identified several Australian *vanB*-type ST78 isolates (collected from 2014 to 2017) (34), and an ST203 isolate that was isolated at another Brisbane hospital in 2016 (35) were closely related to clade 2 isolates, indicating that the common ancestor of clade 2 isolates has likely been circulating in the broader Brisbane region for some time.

MLST profiling is still widely used in clinical settings for epidemiological investigations. However, the discordance between MLST and WGS-based analysis in capturing the structure of VREfm populations has been described previously (37). The limitations of the MLST approach are further highlighted here, where we observed clustering of different ST203 isolates with both of our ST78 clades (clades 1 and 2). These incongruent relationships between STs are likely a result of the highly recombinogenic genome of E. faecium and the localization of genes used to define its MLST in recombination hot spots (37, 38). We also demonstrated the use of SNP-based subtyping to better depict isolate relatedness of ST78 isolates. We used a 20-SNP cutoff, which has been well characterized based on VREfm within-host diversity study (36), to group isolates with minimal recombination impact and isolated more clonally related VREfm. The findings again stress the importance of WGS data for unraveling population structure and evolutionary dynamics of VREfm clones.

Public health surveillance programs routinely investigate locally acquired cases within a global context, particularly in foodborne outbreaks such as Salmonella and Listeria surveillance projects (39). However, due to computational and bioinformatics overheads and a lack of immediate concern in health care settings, this level of scrutiny is rarely applied to HAI. Here, by expanding our reference data set to include a global collection of E. faecium genomes, we found that clade 2 clustered more broadly with ST78 isolates collected from a presumptive outbreak within a UK hospital in 2015 (36). Similar to clade 2, these isolates also demonstrated high endemicity, low genomic diversity, and high acquisition rates in hospital settings. Here, we demonstrate that routine comparisons of locally acquired isolates against global data sets can enhance the infection control response by identifying the infiltration of high-risk clones and triggering a targeted response to prevent these organisms from becoming established. The increased availability of online epidemiological surveillance platforms such as Pathogenwatch (40), NCBI Pathogen Detection (34), and Nextstrain (41) serve to reduce the complexity and technical overheads associated with these types of analysis and provide continuously updated data sets. Leveraging these existing resources will allow for routine comparisons to global data sets to be integrated within HAI surveillance platforms.

Here, the complexity of our transmission networks suggests that multiple modes of transmission have contributed to the spread of the outbreak. In addition to direct transmission between patients, transmission of enterococci from the hospital environment and from health care workers has also been well documented (36, 42, 43). Similar transmission routes are likely at play here, which is highlighted by a lack of clear directionality in our transmission graphs. However, we advise caution when interpreting transmission graphs for bacterial outbreaks as it appears that even in cases in which directionality was predicted with a high degree of certainty, the inferred transmission routes were biased by the collection dates of the samples. Consequently, the predicted directionality is likely to reflect sequential sampling dates rather than a true representation of transmission routes. This potential bias is further supported by the poor correlation between genetic distance (e.g., SNP accumulation) and sampling dates in our data set (Fig. S8). In cases of asymptomatic colonization, admission dates are not an effective data source from which to infer transmission and would likely decrease the accuracy of the prediction (44). Although we attempted to increase the accuracy of our transmission model by including more detailed isolate and patient metadata (e.g., shared wards, lineage subtyping) and adjusting the transmission model into a longer generation and incubation periods, the inclusion of these data had limited impact on our predictions. It should be noted that in most cases, transmission direction was estimated using only the patients’ most recent admission dates. However, regular admission to hospital is not uncommon, particularly among patient cohorts with ongoing or chronic medical conditions (e.g., renal patient undergoing dialysis). For these patients, access to historical admission records would be necessary for accurate reconstruction of transmission networks. Therefore, a model for colonization-based bacterial epidemiology is required for more accurate transmission dynamics, such as the inclusion of bacterial colonization period and distribution, patients’ historical admissions, screenings, and bed movement data into the model of transmission prediction.

Despite repeated attempts, we were unable to confirm broad contamination of the hospital environment with the outbreak strain, the exception being a single environmental isolate collected from a room occupied by a colonized patient. Unfortunately, our sampling strategy focused on admitted patients, and as a result, we were unable to determine whether carriage among hospital staff or visitors was contributing to the spread of the outbreak (43, 45). A recent study by Van Hal et al. (46) described the importance of interactions between E. faecium clones from the community and hospital strains for the ongoing adaptation of VREfm in health care settings. Thus, a more thorough strategy for screening potential carriage and preventing the emergence of high-risk hospital clones could benefit future infection control policies. Additionally, our surveillance project was focused solely on the transmission of antibiotic resistant isolates. Consequently, it is not known whether the silent spread of vancomycin-sensitive E. faecium (VSEfm) (3) or vancomycin-variable E. faecium (VVEfm) (47) among patients has contributed to the extent and longevity of this outbreak. Several high-risk VSEfm clones were cultured from bloodstream infections during the study period; however, the recovered isolates were not related to the outbreak strain (Fig. S9).

Regardless, the application of real-time WGS provided valuable insights into the dynamics and control of this outbreak that will inform future infection control responses. These include outbreak and source control for each identified transmission event, including isolation of the index case under transmission-based precautions, contact screening (immediate patient contacts and whole of ward), enhanced environmental cleaning (bathrooms, high-touch points, and patient care equipment), and environmental hygiene audit and review. Following these WGS-directed interventions, ST78 colonization and infection rates have rapidly decreased, with no new cases identified since June 2021. Interestingly, colonization and infection rates of other clonal lineages remained consistent during and following the resolution of this outbreak, suggesting that WGS may be best used as precision infection control response tool to target resource-limited interventions to control the spread of outbreaks and high-risk clones.

## MATERIALS AND METHODS

### Study setting.

All isolates were collected from a large tertiary referral hospital in southeast Queensland, providing inpatient and outpatient care in all major adult specialties. The hospital has capacity for 1,054 beds with annual 60,908 emergency department (ED) presentations and 110,179 total patient admissions. Surveillance for multidrug-resistant organisms, including vancomycin-resistant Enterococcus (VRE), is performed routinely in high-risk wards, including renal, dialysis, oncology, hematology, intensive care, infectious diseases, transplant services, and both interhospital and international patient transfers. Contact screening was undertaken in response to a positive isolate regardless of colonization or infection.

### Patient and environmental screening of VRE.

Rectal screening swabs were routinely collected from patients at high risk of VRE acquisition (e.g., intensive care, renal dialysis, interhospital transfers). Environmental screening swabs were also taken from high-touch areas from the wards where the patients were identified as VRE positive. The swabs were inoculated into VRE broth and observed for esculin hydrolysis (blackening of the broth) after 24 h (48 h for environmental samples). If this occurred, 10 μL was subcultured onto chromID VRE agar (bioMérieux) and incubated for 48 h at 35°C in air and examined at 24 and 48 h for growth. The presence of violet colonies suggested E. faecium, and the presence of blue-green colonies Enterococcus faecalis. Species level identification was performed using Vitek MS (bioMérieux) and susceptibility testing by automated broth microdilution (Vitek2; bioMérieux). If vancomycin resistance was detected (MIC > 4 mg/liter), isolates were reported as VRE, and if the vancomycin MIC was ≤4 mg/liter, PCR testing for *vanA/B* genes using an in-house real-time PCR assay was performed (details of primers used are given in Table S1). The first VRE isolate from patients newly identified to be colonized or infected with VRE was characterized by whole-genome sequencing. Repeat detection of VRE was not confirmed if it occurred within 3 months of initial isolation, unless further confirmation was required (e.g., clinically significant infection).

### Whole-genome sequencing.

E. faecium isolates from colonization and infection cases were submitted for whole-genome sequencing (WGS) at the Queensland Health Forensic and Scientific Services laboratory. Genomic DNA was extracted using QIAamp DNA minikits (Qiagen, Australia) and quantified by spectrophotometry (Nanodrop; ThermoFisher) and fluorometry (Quant-iT; ThermoFisher). Paired-end DNA libraries were prepared using Nextera XT kits (Illumina; Australia), and WGS was performed using the Illumina NextSeq 500 (150-bp paired end). The sequence reads from this study have been deposited in the Sequence Read Archive (accessions numbers SRR17671373 to SRR17671435). Individual accession numbers and isolate metadata are listed in Table S2.

### *De novo* assemblies and *in silico* genotyping.

The quality of short reads genome sequences was assessed using FastQC v0.11.6 (48). The low-quality bases (<Q20) were filtered using Trimmomatic v0.36 (49). The trimmed reads (median of average base quality of 33.19; Fig. S1) were assembled *de novo* using SPAdes v3.14.0 (50). The quality of the assemblies was assessed using QUAST v5.0.2 (51), and the results are summarized in Table S3. Identification of resistance and virulence genes were performed *in silico* using SRST2 v0.2 (52) against Resfinder 4.0 (53) and the virulence factor database (VFDB) (54). Both databases were manually built from their latest version (access date of 20 January 2021) for compatibility with SRST2. The potential *van*-carrying plasmid replicons were screened from the genome assemblies using Abricate v1.0.1 (55) against Plasmidfinder (56) and BLASTN v2.12 (57) of *vanB* sequences from Resfinder 4.0 (53). Tn*1549*-like transposon sequence was screened from the assemblies against Tn*1549* complete sequence (accession number AF192329) using BLASTN v2.12 (57) and visualized using EasyFig v2.2.2 (58).

### Phylogenetic analysis.

Read mapping and variant calling were performed on the 63 study isolates to extract substitution-only single nucleotide polymorphisms (SNPs) using Lyveset v1.1.4f (59) against E. faecium AUS0085 genome (NCBI RefSeq GCF_000444405.1) (29). Gubbins v2.4.1 (60), bcftools v1.9 (61), snp-sites v2.4.1 (62), and a masking script (63) were used to produce recombination-adjusted core genome SNPs alignment, in which SNPs in recombinant regions have been removed (Fig. S2). The SNPs alignment were used for phylogenetic tree reconstruction using RaxML v8.2.12 (64) with a general-time reversible nucleotide substitution model with gamma correction for site variation and 1,000 bootstrap replicates.

Comparison of study isolates to global E. faecium genomes was performed using Parsnp v1.2 (65). A total of 2,027 E. faecium genomes were available and downloaded from the National Center for Biotechnology Information (NCBI) Genome database (access date 11 November 2020), of which 73 genomes were selected based on their sequence type (ST78 or its single locus variants), *van*-type (*vanB*), and geographical origin (Australia). Metadata for the genomes were retrieved using NCBImeta (66), and the ST and *van*-types were identified using mlst v2.19.0 (67) and Abricate v1.0.1 (55), respectively (Table S4). The extraction of recombination-adjusted core genome SNPs and the reconstruction of the global phylogenetic tree were performed using the aforementioned method.

Whole-genome phylogeny of ST78 with other vancomycin-sensitive E. faecium (VSEfm) was reconstructed using Jolytree v1.1b (68). The phylogenetic tree and metadata were explored and visualized using HAIviz v0.3 (69) and ggtree v2.4.1 (70, 71). Further details are given in the supplemental materials.

### Global cluster analysis.

The cluster relationship between local ST78 with global ST78 and its SLVs was also evaluated by calculating pairwise cgMLST distance using publicly available complete genomes retrieved from NCBI RefSeq and random representative assemblies of E. faecium SNP clusters detected in NCBI Pathogen Detection (access date 10 December 2022) (34). The cgMLST allelic profile was called using Chewbacca v2.8.5 (72) from 1,423 target genes listed by de Been et al. (73) downloaded from https://cgmlst.org. Pairwise cgMLST distances were generated by cgmlst-dists v0.4.0 (74) and were loaded to GraphSNP v1.0 (75). A 25-allele cutoff was used to identify cgMLST clusters, consistent with the NCBI Pathogen wgMLST clustering method (76). The minimum allele differences between identified clusters were visualized using a minimum spanning tree (Fig. S3).

### Subtype analysis.

Genetically close subtypes were investigated using network-based clustering approach implemented in our internal integrated clustering platform CATHAI: Cluster Analysis Tool for Hospital-Associated Infections (77). In brief, a SNP distance cutoff was applied to a network of isolate core genome pairwise SNP distance to identify groups of genetically close isolates represented by a subnetwork referred to as a “subtype.” The approximation of the SNP cutoff was performed based on subtyping described by Gouliouris and colleagues (36). First, the distribution of the pairwise SNP distances before and after recombination filtering was extracted and compared between study isolates (Fig. S4). Next, SNP distances representing minor or no detectable recombination relationships (indicated by a difference between distance with and without recombination ranging from 0 to 50 SNPs) were filtered to determine the subtyping cutoff. Consistent with findings from Gouliouris and colleagues (36), we also found that 20 SNPs were sufficient to reliably represent clonal relationships between E. faecium isolates and thus were selected for our subtyping analysis.

### Transmission analysis.

Outbreaker2 v1.1.1 (44) was used to infer the transmission based on the core genome SNPs, collection dates and distribution of generation and incubation time following a discretized gamma distribution (*k* = 7, θ = 2). Transmission inference was performed using two runs of five million iterations of Monte Carlo Markov Chain (MCMC) with sampling frequency and a burn-in period of 100 and 1,250,000, respectively. Transmission trees with a minimum transmission support of 10% were extracted and integrated with the patient ward sharing data (Fig. S5) and the identified subtypes to produce a combined transmission network. A strong epidemiological link was defined between isolates (representing patients) from the same subtype and collected from patients who had overlapping stays in the same ward or had been admitted to the same ward within 7 days of each other (36). Finally, the transmission network was exported into a DOT-formatted directed graph using R (71) and interactively explored using HAIviz v0.3 (69).

### Ethical approval.

Ethical oversight was provided by the Forensic and Scientific Services Human Ethics Committee (reference HEC17_17) as a low and negligible risk approval, with provision for a waiver of individual patient consent.

### Data availability.

All sequence data derived from this project are publicly available at NCBI Bioproject PRJNA797179. Raw Illumina sequence read data have been deposited in the Sequence Read Archive (accessions numbers SRR17671373 to SRR17671435; Table S2).
