# Supplementary material for: Using Genomics To Investigate an Outbreak of Vancomycin-Resistant Enterococcus faecium ST78 at a Large Tertiary Hospital in Queensland
Source: Microbiol Spectr. 2023 May 16;11(3):e04204-22. doi: 10.1128/spectrum.04204-22 (PMC10269735; doi:10.1128/spectrum.04204-22)
Supplement: Supplemental file 1 — Supplemental methods, Tables S1 to S9, and Fig. S1 to S9. Download spectrum.04204-22-s0001.docx, DOCX file, 1.4 MB [file spectrum.04204-22-s0001.docx]

Supplementary Information (SI)

Using genomics to investigate the epidemiology of Vancomycin-resistant *Enterococcus faecium* ST78 at a large tertiary hospital in Queensland

**Budi Permana**^1,2^, Patrick N. A. Harris^3,4,5^, Naomi Runnegar^6,7^, Margaret Lindsay^7^, Belinda C. Herderson^7^. Geoffrey E. Playford^7^, David Paterson^4^, Scott A. Beatson^1,2,3^, Brian M. Forde^3,4*^

^1^ School of Chemistry and Molecular Biosciences, Faculty of Science, The University of Queensland, Brisbane, Australia

^2^ Australian Centre for Ecogenomics, The University of Queensland, Brisbane, Australia

^3^ Australian Infectious Disease Research Centre, Faculty of Science, The University of Queensland, Brisbane, Australia

^4^ UQ Centre for Clinical Research, Faculty of Medicine, The University of Queensland, Brisbane, Australia

^5^ Pathology Queensland, Central Laboratory, Brisbane, Australia

^6^ PA-Southside Clinical School, Faculty of Medicine, The University of Queensland, Brisbane, Australia

^7^ Infection Management Services, Princess Alexandra Hospital, Brisbane, Australia

^8^ Herston Infectious Diseases Institute, Metro North Health, Brisbane, Australia

* Corresponding authors:

**Brian M. Forde** ([b.forde@uq.edu.au](mailto:b.forde@uq.edu.au))

**Supplementary methods**

**Reads quality control, de novo assembly and *in* *silico* genotyping**

The quality of short-reads genome sequences was assessed using FastQC v0.11.6 (1). Low-quality bases (<Q20) were filtered using Trimmomatic v0.36 (2). Passed-quality control short reads that with the median of average base quality of 33.19 (IQR 32.65–33.51) (Supplementary figure S4.1) were *de novo* assembled using SPAdes v3.14.0 (3). The quality of assemblies was assessed using QUAST v5.0.2 (4). Identification of resistance and virulence genes were performed *in silico* using SRST2 v0.2 (5) under the default settings against Resfinder 4.0 database (6) and virulence factor database (VFDB) (7), respectively. Both databases were manually built from their latest version per access date of 20-Jan-2021 for compatibility purposes with SRST2 (as instructions described in <https://github.com/katholt/srst2>). Detected sequences were tabulated to the level of the genes, and the present and absent genes were inspected and visualised.

**Phylogenetic analysis**

A total of 7,553 single nucleotide polymorphism (SNP) sites were identified from read mapping of 63 isolates against a complete genome of *E. faecium* AUS0085 (accession number ASM44440v1) using combination of SMALT (8) and VarScan (9) implemented in Lyveset v1.1.4f (10) under the default setting (minimum for coverage and alternate fraction are 10 and 75%, respectively). SNPs from mobile genetic elements (MGEs) region were filtered out leaving 5,838 sites defined as core genome SNPs. The SNPs used to create a pseudo whole genomes alignment relative to the reference using BCFTools consensus (11) then was used to identify recombination events using Gubbins v.2.4.1 (12). The identified putative recombination regions were masked from the alignment using a masking script (<https://github.com/kwongj/cfml-maskrc)> (Supplementary figure S4.2). A total of 344 non-ambiguous SNPs were extracted using snp-sites –c (13) and defined as recombination-adjusted core genome SNPs. These final SNPs were used to build a maximum likelihood phylogenetic tree using RaxML v.8.2.12 (14) with a general-time reversible nucleotide substitution model with GAMMA correction for site variation. One thousand bootstrap replicates were performed to evaluate the branch support within the phylogenetic tree. The final tree and metadata were explored and visualised using HAIviz v.0.3 (15) and ggtree R package (16, 17)**.**

**Regional and global context of our study isolates**

To evaluate where our study isolates fit within the regional and global *E. faecium* ST78 population, publicly available genomes were downloaded from the NCBI Genome database (accessed on 11-11-2020), and the metadata was retrieved using NCBImeta (18). A total of 2027 genomes were identified and retrieved, consisting of 147 complete genomes, 16 chromosomes, 1020 contigs, and 844 scaffolds. *In silico* multi-locus sequence, typing was performed to screen ST78 using mlst tool v2.19.0 (<https://github.com/tseemann/mlst>), resulting in 67 ST78 genomes (Australia: 1, Brazil: 1, China: 13, Japan: 1, New Zealand: 1, Russia: 7, USA: 3, Unknown: 3, and European country: 37). Due to a lower number of Australian samples, to increase the resolution of the regional context, we also included other samples collected from Australia that identified as *vanB-*type or a single locus variant to ST78. A total of 73 public *E. faecium*genomes were included (Supplementary table S2) and used to create core genomes alignment relative to AUS0085 (accession number ASM44440v1) using ParSNP v1.2 from Harvest suite (19) with the default setting. Recombination-adjusted core genome SNPs were extracted and used to build phylogeny using the same phylogenetic analysis approach described in the previous section. The final tree and the associated metadata were visualised using ggtree v2.4.1 R package (16, 17).

**Subtype analysis**

Genetically close subtypes were investigated using a network-based clustering approach implemented in our internal integrated clustering platform CATHAI: Cluster Analysis Tool for Hospital-Associated Infections (20). In brief, an SNP distance cut-off was applied to a network of isolate’s core genome pairwise SNP distances to identify the genetically close group of isolates represented by a sub-network referred here as “subtype”. First, the approximation of the SNPs cut-off was performed based on sub-typing described by Gouliouris and colleagues (21). Then, the distribution of the pairwise SNP distances with and without the recombination sites was compared to identify a group of isolates that had minor or no detectable recombination (the difference of SNPs with and without recombination is ranging from 0-50 SNPs) under the assumption that this group was more likely to be an outbreak-associated subtype. The results showed that the 20 SNPs threshold includes the majority of isolates in this group either in core SNPs with or without recombination (Supplementary figure S4.3). Thus, a cut-off of 20 core genome SNPs was used in our subtype analysis.

**Transmission analysis**

Outbreaker2 (22) was used to infer the base transmission trees based on the core genome SNPs, collection dates, and distribution of generation and incubation time following a discretised-Gamma distribution (k = 7, θ =2). Briefly, tree inference was performed using two runs of five million iterations of Monte Carlo Markov Chain (MCMC) with sampling frequency and a burn-in period of 100 and 1,250,000, respectively. First, the convergence of MCMC was assessed using the coda R package (23) and the consensus of transmission trees was extracted with the transmission support of a minimum 10%. Next, genomic and epidemiological relationships from subtype analysis and patient ward sharing data were added into the inferred transmission trees using R (17), producing a combined transmission network. A strong epidemiological link was defined between isolates (which are representing the patients) from the same subtype and was collected from patients who had ward overlap (both patients had stayed in the same ward at the same time) or had ward gap of up to 7 days (patients stayed at the same ward but at different times no longer than seven days) (21). Finally, the final transmission network was exported into a DOT-formatted directed graph for interactive visualisation and collective evaluation with the metadata on HAIviz v0.3 (15).

**Interactive visualisation of Queensland VREfm ST78 subtypes using GraphSNP**

Interactive visualisation of Queensland VREfm ST78 subtypes was performed and is provided in GraphSNP v1.0. Readers can recreate, view, and explore these subtypes using the following steps:

1. Navigate to the GraphSNP website (<https://graphsnp.fordelab.com/graphsnp>) and select preloaded dataset titled “Using genomics to investigate the epidemiology of Vancomycin-resistant *Enterococcus faecium* ST78 at a large tertiary hospital in Queensland.
2. Create graph with the specified settings: *Type of analysis: Clustering; Method: CATHAI; Cut-off: 20; Node color: ward.*

Additional settings such as network layout, node size, and text labels can be adjusted for enhanced visualisation.

**Supplementary results**

**All study isolates carried virulence genes commonly found in clinically significant VREfm**

To investigate the potential association between virulence and the outbreak, putative virulence factors were screened from the study isolates. Fourteen virulence genes were identified, with nine of which were present in all isolates (Supplementary table S8). These include gene *esp*, which encodes enterococcal surface protein (24), genes *acm* (25), *sgrA* *(26)*, *scm* (27), and *efaA*, which encodes microbial surface components recognizing adhesive matrix molecules (MSCRAMM), gene *bopD* that promote the host cells invasion and biofilm formation (28), genes *uppS* and *sagA*, which have been implicated in cell wall biosynthesis and metabolism *(29)*, and gene EFAU085_01747 encoding phosphatidate cytidylyltransferase (30). These genes have been frequently reported in clinical *E. faecium* isolates (24, 31, 32). An additional five virulence genes, including *ebpB* and genes encoding pili: *pilA*, *pilB*, *pilE*, and *pilF* (33), were also detected but mainly present in clade 1 isolates. Overall, these genes were present in most isolates and were not associated with the major outbreak subtypes in clade 2 (Supplementary Figure S2).

**Supplementary tables**

**Supplementary Table S1:** VanA/B PCR primers

| Primer | Sequence | Length |
| --- | --- | --- |
| VANAF | GGGAAACAGTGCCGCGT | 17 bp |
| VANBF | GTCATGGGGAACGAGGATGA | 20 bp |
| VANABUNIR | TCCGGCTCGACTTCCTGAT | 19 bp |

**Supplementary Table S2:** Metadata of 63 VREfm ST78 isolates used in this study

| Isolate ID | Accession number | Patient ID | Collection date | Collection location | Collection type | Clade group | Subtype |
| --- | --- | --- | --- | --- | --- | --- | --- |
| M86159 | SRR17671435 | P-1 | 2018-06-30 | L5-WK | Rectal swab | 2 | 2B |
| M810154 | SRR17671434 | P-2 | 2018-11-07 | L4-WF | Rectal Swab | 2 | 2B |
| M810885 | SRR17671423 | P-3 | 2018-11-28 | L5-WK | Rectal Swab | 1 | Singleton |
| M91414 | SRR17671412 | P-4 | 2019-01-24 | L3-WQ | Rectal Swab | 2 | 2C |
| M93370 | SRR17671401 | P-5 | 2019-03-12 | L4-WE | Rectal Swab | 1 | 1D |
| M93924 | SRR17671390 | P-6 | 2019-03-23 | L4-WE | Rectal Swab | 1 | 1D |
| M94211 | SRR17671379 | P-7 | 2019-03-31 | L4-WF | Rectal Swab | 2 | 2B |
| M94926 | SRR17671375 | P-8 | 2019-04-21 | L2-WD | Rectal Swab | 2 | 2B |
| M94927 | SRR17671374 | P-9 | 2019-04-23 | L2-WD | Rectal Swab | 1 | Singleton |
| M95768 | SRR17671373 | P-10 | 2019-05-18 | L1-WB | Rectal Swab | 2 | 2A |
| M96011 | SRR17671433 | P-11 | 2019-05-22 | LHC-WP | Rectal Swab | 2 | Singleton |
| M96902 | SRR17671432 | P-12 | 2019-06-16 | L4-WH | Rectal Swab | 2 | 2B |
| M97750 | SRR17671431 | P-13 | 2019-07-16 | L3-WM | Urine | 2 | 2A |
| M97944 | SRR17671430 | P-14 | 2019-07-21 | L5-WK | Rectal Swab | 2 | 2A |
| M97945 | SRR17671429 | P-15 | 2019-07-22 | L5-WK | Rectal Swab | 2 | 2A |
| M98146 | SRR17671428 | P-16 | 2019-07-26 | L2-WC | Humerus Tissue | 2 | 2A |
| M98147 | SRR17671427 | P-17 | 2019-07-30 | L2-WD | Rectal Swab | 2 | 2A |
| M98516 | SRR17671426 | P-18 | 2019-08-17 | L4-WF | Rectal Swab | 2 | 2A |
| M98703 | SRR17671425 | P-19 | 2019-08-19 | LSI-WR | Rectal Swab | 2 | 2A |
| M98889 | SRR17671424 | P-20 | 2019-08-26 | L4-WF | Rectal Swab | 2 | 2A |
| M98884 | SRR17671422 | P-21 | 2019-08-27 | L2-WD | Rectal Swab | 2 | 2A |
| M99250 | SRR17671421 | P-22 | 2019-09-09 | L3-WQ | Rectal Swab | 2 | 2A |
| M910050 | SRR17671420 | P-18 | 2019-09-17 | L5-WI | Blood | 2 | 2A |
| M99402 | SRR17671419 | P-24 | 2019-09-22 | L3-WQ | Rectal Swab | 2 | 2A |
| M99624 | SRR17671418 | P-25 | 2019-09-26 | L4-WH | Anal Swab | 2 | Singleton |
| M99825 | SRR17671417 | P-26 | 2019-10-02 | LGA-WL | Skull Bone | 2 | 2A |
| M99826 | SRR17671416 | P-27 | 2019-10-03 | L3-WQ | Rectal Swab | 2 | 2C |
| M910882 | SRR17671415 | P-28 | 2019-10-22 | L5-WJ | Rectal Swab | 2 | 2C |
| M910479 | SRR17671414 | P-29 | 2019-10-23 | L5-WK | Rectal Swab | 2 | 2A |
| M910478 | SRR17671413 | P-30 | 2019-10-23 | L4-WF | Rectal Swab | 2 | 2A |
| M910480_2 | SRR17671411 | P-16 | 2019-10-24 | L5-WK | Humerus Tissue | 2 | 2A |
| M910665_1 | SRR17671410 | P-32 | 2019-10-29 | L2-WD | Rectal Swab | 2 | 2A |
| M910881 | SRR17671409 | P-33 | 2019-10-30 | L4-WF | Rectal Swab | 2 | 2A |
| M910888 | SRR17671408 | P-34 | 2019-11-04 | L3-WQ | Rectal Swab | 2 | 2A |
| M911142 | SRR17671407 | P-35 | 2019-11-07 | L4-WF | Rectal Swab | 2 | 2A |
| M911374 | SRR17671406 | P-36 | 2019-11-07 | LHD-WO | Rectal Swab | 2 | 2A |
| M911146 | SRR17671405 | P-37 | 2019-11-12 | L2-WD | Rectal Swab | 2 | 2A |
| M911140 | SRR17671404 | E-1 | 2019-11-12 | L5-WK | Environmental Swab | 2 | 2A |
| M911144 | SRR17671403 | P-39 | 2019-11-14 | L3-WQ | Rectal Swab | 2 | 2A |
| M911362 | SRR17671402 | P-40 | 2019-11-18 | L4-WG | Rectal Swab | 2 | 2A |
| M911373 | SRR17671400 | P-41 | 2019-11-18 | L4-WG | Rectal Swab | 2 | 2A |
| M911375 | SRR17671399 | P-42 | 2019-11-18 | L3-WQ | Rectal Swab | 2 | 2A |
| M911364 | SRR17671398 | P-43 | 2019-11-21 | L2-WC | Foot Tissue | 2 | 2A |
| M911620 | SRR17671397 | P-44 | 2019-11-25 | L4-WF | Rectal Swab | 2 | 2A |
| M911619 | SRR17671396 | P-45 | 2019-11-27 | L3-WQ | Rectal Swab | 2 | 2A |
| M911842 | SRR17671395 | P-46 | 2019-12-06 | L5-WK | Rectal Swab | 2 | 2A |
| M912102 | SRR17671394 | P-47 | 2019-12-11 | L4-WF | Rectal Swab | 2 | 2B |
| M912106 | SRR17671393 | P-48 | 2019-12-11 | L4-WF | Rectal Swab | 2 | 2B |
| M912103 | SRR17671392 | P-49 | 2019-12-11 | L2-WC | Rectal Swab | 2 | 2A |
| M00066 | SRR17671391 | P-50 | 2019-12-16 | L4-WH | Rectal Swab | 2 | 2C |
| M00065 | SRR17671389 | P-51 | 2019-12-16 | L4-WH | Rectal Swab | 2 | Singleton |
| M00363 | SRR17671388 | P-52 | 2019-12-31 | L1-WA | Rectal Swab | 2 | 2A |
| M00362 | SRR17671387 | P-53 | 2020-01-01 | L4-WF | Rectal Swab | 2 | 2B |
| M00680 | SRR17671386 | P-54 | 2020-01-05 | L4-WF | Rectal Swab | 2 | 2A |
| M00943 | SRR17671385 | P-55 | 2020-01-08 | L4-WF | Rectal Swab | 2 | 2B |
| M00945 | SRR17671384 | P-56 | 2020-01-12 | L4-WF | Rectal Swab | 2 | 2A |
| M01235 | SRR17671383 | P-57 | 2020-01-15 | LHD-WO | Rectal Swab | 2 | 2A |
| M01236 | SRR17671382 | P-58 | 2020-01-16 | L4-WF | Rectal Swab | 1 | Singleton |
| M01234 | SRR17671381 | P-59 | 2020-01-17 | L5-WK | Rectal Swab | 2 | 2A |
| M01603 | SRR17671380 | P-60 | 2020-01-24 | L1-WA | Rectal Swab | 2 | 2A |
| M05847 | SRR17671378 | P-61 | 2020-05-10 | L3-WQ | Rectal Swab | 2 | Singleton |
| M06114_3 | SRR17671377 | P-62 | 2020-06-25 | L1-WN | Blood | 2 | 2A |
| M07824 | SRR17671376 | P-63 | 2020-09-21 | L5-WK | Rectal Swab | 2 | 2A |

**Supplementary Table S3:** Quality metrics for genome assemblies

| **Assembly** | **Number of contigs** | **Largest contig** | **Total length** | **GC (%)** | **N50** |
| --- | --- | --- | --- | --- | --- |
| M910050 | 486 | 48751 | 2646979 | 38.51 | 10150 |
| M01603 | 614 | 46647 | 2850395 | 38.22 | 11045 |
| M98146 | 415 | 65011 | 2804112 | 38.17 | 14108 |
| M910480_2 | 370 | 62969 | 2763393 | 38.27 | 16267 |
| M99624 | 369 | 75879 | 2793704 | 38.25 | 16458 |
| M00066 | 401 | 51150 | 2876006 | 38.26 | 16608 |
| M98703 | 370 | 59445 | 2804831 | 38.15 | 17107 |
| M98889 | 352 | 75054 | 2756380 | 38.29 | 17735 |
| M98884 | 399 | 72042 | 2883431 | 38.06 | 18659 |
| M910888 | 365 | 73584 | 2844935 | 38.04 | 18758 |
| M00680 | 350 | 59320 | 2802119 | 38.15 | 18978 |
| M00065 | 352 | 68062 | 2857185 | 38.1 | 19209 |
| M910478 | 392 | 66631 | 2870476 | 38.1 | 19541 |
| M910882 | 448 | 67855 | 2976385 | 38.14 | 20474 |
| M98516 | 363 | 71354 | 2848489 | 38.04 | 20845 |
| M97750 | 400 | 66211 | 2866346 | 38.02 | 21132 |
| M97944 | 364 | 73108 | 2849860 | 38.03 | 21254 |
| M910479 | 419 | 67286 | 2873368 | 38 | 21254 |
| M96011 | 320 | 74056 | 2836482 | 38.04 | 22513 |
| M00362 | 394 | 78569 | 2974845 | 37.98 | 22559 |
| M911619 | 310 | 81442 | 2835680 | 38.05 | 22776 |
| M911620 | 352 | 63568 | 2899028 | 37.99 | 23396 |
| M911842 | 384 | 77821 | 2923564 | 37.96 | 23889 |
| M97945 | 351 | 79233 | 2837104 | 38.05 | 23940 |
| M912103 | 297 | 86560 | 2789024 | 38.13 | 23998 |
| M810154 | 323 | 85806 | 2952041 | 37.93 | 25606 |
| M86159 | 298 | 119233 | 2775014 | 38.18 | 25615 |
| M91414 | 432 | 93165 | 3038837 | 37.9 | 25615 |
| M99402 | 420 | 87043 | 2895113 | 37.93 | 26721 |
| M99250 | 357 | 74055 | 2933515 | 37.92 | 27782 |
| M99826 | 372 | 87755 | 2952857 | 38.1 | 27897 |
| M98147 | 330 | 79317 | 2859225 | 37.99 | 27967 |
| M910881 | 369 | 77821 | 2971359 | 37.88 | 28009 |
| M911140 | 290 | 92661 | 2842348 | 37.99 | 28017 |
| M00945 | 279 | 86582 | 2776001 | 38.12 | 28020 |
| M94926 | 381 | 115572 | 3061382 | 37.82 | 28049 |
| M911364 | 346 | 86632 | 2944137 | 37.88 | 28085 |
| M00363 | 334 | 80715 | 2880668 | 37.97 | 28154 |
| M912106 | 291 | 93615 | 2939228 | 37.97 | 28586 |
| M912102 | 279 | 94186 | 2937171 | 37.97 | 28607 |
| M911373 | 308 | 86564 | 2765410 | 38.16 | 28712 |
| M05847 | 296 | 119365 | 2930322 | 37.97 | 28778 |
| M810885 | 354 | 90706 | 2923993 | 38.04 | 29044 |
| M911146 | 283 | 74055 | 2865627 | 37.96 | 29142 |
| M01235 | 344 | 86582 | 2886197 | 37.95 | 29376 |
| M911142 | 375 | 87093 | 3012036 | 37.74 | 29376 |
| M01234 | 293 | 85538 | 2857093 | 37.97 | 29682 |
| M911144 | 305 | 86572 | 2871442 | 37.94 | 29682 |
| M99825 | 341 | 87093 | 2936948 | 37.91 | 29682 |
| M911374 | 339 | 78008 | 2885410 | 37.93 | 29683 |
| M911362 | 268 | 86582 | 2767633 | 38.14 | 29741 |
| M00943 | 291 | 84834 | 2979641 | 37.84 | 30333 |
| M95768 | 316 | 77916 | 2877241 | 37.92 | 30899 |
| M06114_3 | 272 | 86947 | 2897578 | 37.93 | 31015 |
| M911375 | 292 | 86572 | 2871245 | 37.93 | 31391 |
| M94211 | 251 | 83437 | 2800289 | 38.08 | 31756 |
| M96902 | 283 | 89403 | 2996886 | 37.83 | 31894 |
| M07824 | 280 | 92258 | 2904178 | 37.89 | 34594 |
| M910665_1 | 320 | 92258 | 2960663 | 37.85 | 35016 |
| M93924 | 369 | 138917 | 2919488 | 37.94 | 43674 |
| M93370 | 258 | 126064 | 2883732 | 38.02 | 44753 |
| M01236 | 205 | 152646 | 2816360 | 38.08 | 47796 |
| M94927 | 269 | 127046 | 2870559 | 38.01 | 48771 |

**Supplementary Table S4:** Public genomes metadata used in regional and global context analysis

| Isolate id | Collection location | Source type | Van type (Abricate results) | ST (mlst results) | Year |
| --- | --- | --- | --- | --- | --- |
| VREfm_AUS0085_ref | Australia (Victoria) | Clinical | VanB | 203 | 2009 |
| GCF_012070685.1 | China | Clinical | VanM | 78 | 2018 |
| GCF_012070665.1 | China | Clinical | VanM | 78 | 2018 |
| GCF_012062555.1 | China | Clinical | VanM | 78 | 2016 |
| GCF_013179755.1 | China | Clinical | VanM | 78 | 2018 |
| GCF_010120755.1 | China | Clinical | VanM | 78 | 2017 |
| GCF_009938075.1 | China | Clinical | VanM | 78 | 2017 |
| GCF_009791335.1 | Portugal | Clinical | VanA | 78 | 2012 |
| GCF_009733985.1 | China | Clinical | VanM | 78 | 2017 |
| GCF_009733995.1 | China | Clinical | #N/A | 78 | 2018 |
| GCF_009734005.1 | China | Clinical | VanM | 78 | 2017 |
| GCF_008974665.2 | Japan | Clinical | VanA | 78 | unknown |
| GCF_000328425.1 | China | Clinical | #N/A | 78 | 2006 |
| GCF_000322425.1 | Denmark | Dog | #N/A | 78 | unknown |
| GCF_000322405.1 | Netherlands | Clinical | #N/A | 78 | 2006 |
| GCF_006375635.1 | Germany | Bird | VanA | 78 | 2011 |
| GCF_006007925.1 | China | Clinical | VanA | 78 | 2018 |
| GCF_001481405.1 | Unknown | Clinical | VanA | 78 | 2015 |
| GCF_005234875.1 | New Zealand | Clinical | VanA | 78 | 2009 |
| GCF_900639425.1 | Unknown | Clinical | VanA | 78 | unknown |
| GCF_900638805.1 | Unknown | Dog | #N/A | 78 | unknown |
| GCF_004152505.1 | USA | Clinical | VanA | 78 | 2018 |
| GCF_004152185.1 | USA | Clinical | #N/A | 78 | 2018 |
| GCF_004152205.1 | USA | Clinical | VanA | 78 | 2018 |
| GCF_900180175.1 | United Kingdom | Clinical | VanA | 78 | 2015 |
| GCF_900178875.1 | United Kingdom | Clinical | VanA | 78 | 2015 |
| GCF_900178845.1 | United Kingdom | Clinical | VanA | 78 | 2015 |
| GCF_900178745.1 | United Kingdom | Clinical | VanA | 78 | 2015 |
| GCF_900178765.1 | United Kingdom | Clinical | VanA | 78 | 2015 |
| GCF_900179915.1 | United Kingdom | Clinical | #N/A | 78 | 2015 |
| GCF_900179295.1 | United Kingdom | Clinical | VanA | 78 | 2015 |
| GCF_900179085.1 | United Kingdom | Clinical | VanA | 78 | 2015 |
| GCF_900179055.1 | United Kingdom | Clinical | VanA | 78 | 2015 |
| GCF_900178625.1 | United Kingdom | Clinical | VanA | 78 | 2015 |
| GCF_900178935.1 | United Kingdom | Clinical | VanA | 78 | 2015 |
| GCF_900178725.1 | United Kingdom | Clinical | VanA | 78 | 2015 |
| GCF_900178985.1 | United Kingdom | Clinical | VanA | 78 | 2015 |
| GCF_900178595.1 | United Kingdom | Clinical | VanA | 78 | 2015 |
| GCF_900178755.1 | United Kingdom | Clinical | VanA | 78 | 2015 |
| GCF_900178555.1 | United Kingdom | Clinical | VanA | 78 | 2015 |
| GCF_900178915.1 | United Kingdom | Clinical | VanA | 78 | 2015 |
| GCF_900178905.1 | United Kingdom | Clinical | VanA | 78 | 2015 |
| GCF_900178975.1 | United Kingdom | Clinical | VanA | 78 | 2015 |
| GCF_900178855.1 | United Kingdom | Clinical | VanA | 78 | 2015 |
| GCF_900178835.1 | United Kingdom | Clinical | VanA | 78 | 2015 |
| GCF_900178785.1 | United Kingdom | Clinical | VanA | 78 | 2015 |
| GCF_900178715.1 | United Kingdom | Clinical | VanA | 78 | 2015 |
| GCF_900178815.1 | United Kingdom | Clinical | VanA | 78 | 2015 |
| GCF_900178925.1 | United Kingdom | Clinical | VanA | 78 | 2015 |
| GCF_900179785.1 | United Kingdom | Clinical | VanA | 78 | 2015 |
| GCF_003719365.1 | China | Clinical | VanA | 78 | 2015 |
| GCF_003332845.1 | Brazil | Clinical | VanA | 78 | 2005 |
| GCF_003284825.1 | Russia | Clinical | VanA | 78 | 2015 |
| GCF_003284815.1 | Russia | Clinical | VanA | 78 | 2015 |
| GCF_003240455.1 | Russia | Clinical | VanA | 78 | 2012 |
| GCF_002848385.1 | China | Livestock fodder | VanA | 78 | 2016 |
| GCF_002562805.1 | Russia | Clinical | VanB | 78 | 2012 |
| GCF_002630975.1 | Russia | Clinical | #N/A | 78 | 2012 |
| GCF_002630985.1 | Russia | Clinical | #N/A | 78 | 2012 |
| GCF_002009635.1 | Russia | Clinical | #N/A | 78 | 2011 |
| GCF_001545685.1 | Sweden | Clinical | VanB | 78 | 2014 |
| GCF_000172915.1 | Netherlands | Clinical | #N/A | 78 | 2005 |
| GCF_000322465.1 | Portugal | Clinical | #N/A | 78 | 2010 |
| GCF_000322445.1 | Latvia | Clinical | #N/A | 78 | 2010 |
| GCF_000322385.1 | Hungary | Clinical | VanA | 78 | 2005 |
| GCF_000322365.1 | Germany | Clinical | #N/A | 78 | 2002 |
| GCF_000322345.1 | Italy | Clinical | #N/A | 78 | 1999 |
| GCF_009769955.1 | Australia (Tasmania) | Clinical | VanB | 796 | 2016 |
| GCF_900092475.1 | Australia (Victoria) | Clinical | VanB | 796 | 2011 |
| GCF_003957785.1 | Australia (Brisbane) | Clinical | VanB | 203 | 2016 |
| GCF_003020705.1 | Australia (Victoria) | Clinical | VanA | 78 | 2015 |
| GCF_003020725.1 | Australia (Victoria) | Clinical | VanB | 796 | 2015 |
| GCF_003020745.1 | Australia (Victoria) | Clinical | VanA | 203 | 2015 |

**Supplementary Table S5:** Location of *vanB* gene in genome assemblies

| Isolate ID | Contig’s IDs where putative plasmid replicons were found (coverage and identity >= 80%) | Contig’s IDs where *VanB* gene sequence was found (blastn identity >= 99%) | *vanB* found in the same plasmid contig ID |
| --- | --- | --- | --- |
| M93370 | 70;72;85;100;110;112;131 | 15;45 | FALSE |
| M93924 | 91;119;130;146;161;166;195;220 | 36;60;553 | FALSE |
| M00363 | 80;116;127;147 | 21;151 | FALSE |
| M00680 | 96;135;155;212 | 9;185 | FALSE |
| M00945 | 80;106;112;150;154;162 | 14;138 | FALSE |
| M01234 | 78;103;111;124;161 | 18;137 | FALSE |
| M01235 | 63;79;104;157;160;164 | 17;131 | FALSE |
| M01603 | 73;154;157;203 | 3;245 | FALSE |
| M06114_3 | 85;106;109;114;117;150 | 13;134 | FALSE |
| M07824 | 69;96;103;108;109;139 | 18;126 | FALSE |
| M910050 | 2;58;147;748 | 156;250 | FALSE |
| M910478 | 84;177;196;244;246 | 18;198 | FALSE |
| M910479 | 87;131;149;200;211;216 | 14;176 | FALSE |
| M910480_2 | 77;131;156;247 | 11;201 | FALSE |
| M910665_1 | 71;91;93;103;105;144 | 18;124 | FALSE |
| M910881 | 80;122;126;153;186;204 | 17;154 | FALSE |
| M910888 | 78;141;164;176;212;222 | 8;184 | FALSE |
| M911140 | 81;113;124;139;167 | 13;146 | FALSE |
| M911142 | 81;93;110;117;122;171;173 | 18;147 | FALSE |
| M911144 | 80;117;128;135;151;157 | 17;136 | FALSE |
| M911146 | 86;122;139;150;155;164 | 16;140 | FALSE |
| M911362 | 76;103;116;156;164;166 | 17;136 | FALSE |
| M911364 | 80;112;120;126;171;173 | 16;151 | FALSE |
| M911373 | 79;104;110;120;160;163 | 15;143 | FALSE |
| M911374 | 80;110;120;149;156;162 | 17;139 | FALSE |
| M911375 | 78;101;109;114;145;150 | 16;132 | FALSE |
| M911619 | 89;126;140;185;195;220 | 17;161 | FALSE |
| M911620 | 91;131;146;173;211;212 | 15;174 | FALSE |
| M911842 | 81;135;156;172;200;202 | 12;173 | FALSE |
| M912103 | 86;130;161;179;187;189 | 12;159 | FALSE |
| M95768 | 80;103;110;114;155 | 18;132 | FALSE |
| M97750 | 88;149;176;195;207;227 | 18;174 | FALSE |
| M97944 | 93;131;160;198;205;217 | 14;172 | FALSE |
| M97945 | 82;122;138;151;193 | 19;163 | FALSE |
| M98146 | 75;145;176;265;296 | 9;212 | FALSE |
| M98147 | 79;113;125;175;185 | 15;149 | FALSE |
| M98516 | 96;151;181;199;207;224 | 12;179 | FALSE |
| M98703 | 85;141;160;185;243 | 11;200 | FALSE |
| M98884 | 100;145;157;188;219;229 | 24;189 | FALSE |
| M98889 | 86;149;190;231;241 | 14;192 | FALSE |
| M99250 | 82;122;153;178;181;189 | 17;154 | FALSE |
| M99402 | 84;120;131;164;172;183 | 13;152 | FALSE |
| M99825 | 81;109;121;138;170;171 | 16;147 | FALSE |
| M00362 | 65;79;128;159;234 | 12;188 | FALSE |
| M00943 | 68;110;121;144;146;157 | 18;143 | FALSE |
| M810154 | 70;93;114;132;163;191 | 15;161 | FALSE |
| M86159 | 64;114;128 | 17;158 | FALSE |
| M912102 | 70;90;119;141;177 | 16;157 | FALSE |
| M912106 | 69;88;117;132;186 | 16;160 | FALSE |
| M94211 | 58;66;114;165 | 18;129 | FALSE |
| M94926 | 67;109;119;129;148;165 | 16;162 | FALSE |
| M96902 | 72;89;105;129;138 | 19;135 | FALSE |
| M00066 | 84;177;203;251 | 10;219 | FALSE |
| M910882 | 78;149;168;177;211;234 | 12;181 | FALSE |
| M91414 | 87;116;129;136;150;161;179 | 14;159 | FALSE |
| M99826 | 87;132;140;146;155;179 | 16;160 | FALSE |
| M01236 | 27;67;92 | 9;16 | FALSE |
| M810885 | 83;103;124;154;169;227 | 11;62 | FALSE |
| M94927 | 68;69;90;96 | 10 | FALSE |
| M00065 | 72;148;192;198;260 | 12;187 | FALSE |
| M05847 | 20;56;70;137;146;157 | 16;150 | FALSE |
| M96011 | 73;129;145;172;197 | 16;176 | FALSE |
| M99624 | 89;153;175;219;252;290;291 | 10;188 | FALSE |

**Supplementary Table S6:** List of resistance genes identified based on *in silico* genotyping

| Gene | AMR Class | Presentation in isolates (%) | | |
| --- | --- | --- | --- | --- |
|  |  | Clade 1 | Clade 2 | Overall |
| *vanHBX* | Glycopeptide | 100.00 | 100.00 | 100.00 |
| *aac(6')-aph(2'')* | Aminoglycoside | 80.00 | 77.60 | 77.80 |
| *aac(6')-Ii* | Aminoglycoside | 100.00 | 100.00 | 100.00 |
| *ant(6)-Ia* | Aminoglycoside | 0.00 | 6.90 | 6.30 |
| *aph(2'')-Ie* | Aminoglycoside | 0.00 | 70.69 | 65.08 |
| *aph(3')-III* | Aminoglycoside | 100.00 | 0.00 | 7.94 |
| *dfrG* | Folate pathway antagonist | 0.00 | 100.00 | 92.06 |
| *erm(B)* | Macrolide, Lincosamide, Streptogramin B | 100.00 | 6.90 | 14.29 |
| *erm(T)* | Macrolide, Lincosamide, Streptogramin B | 0.00 | 70.69 | 65.08 |
| *lnu(B)* | Lincosamide | 0.00 | 6.90 | 6.35 |
| *lsa(E)* | Lincosamide, Streptogramin A, Pleuromutilin | 0.00 | 6.90 | 6.35 |
| *msr(C)* | Macrolide, Streptogramin B | 100.00 | 100.00 | 100.00 |
| *tet(L)* | Tetracycline | 0.00 | 94.83 | 87.30 |
| *tet(M)* | Tetracycline | 100.00 | 100.00 | 100.00 |

**Supplementary Table S7:** Statistics of pairwise SNP distances within clade group

| Category | Group | Clade | Number of isolates | Pairwise SNP distances | | | | | |
| --- | --- | --- | --- | --- | --- | --- | --- | --- | --- |
|  |  |  |  | Min | Q1 | Median | Mean | Q3 | Max |
| Before recombination filtering (core genome SNPs) | Within group | 1 | 5 | 0 | 29.5 | 39 | 33.4 | 41.75 | 42 |
|  |  | 2 | 58 | 0 | 3 | 12 | 99.7 | 231 | 314 |
|  | Between group | 1 & 2 | 63 | 1244 | 1348 | 1352 | 1381 | 1419 | 1514 |
|  | Overall | 1 & 2 | 63 | 0 | 3 | 164 | 289 | 238 | 1514 |
| **After recombination** **filtering** (recombination-adjusted core genome SNPs) | Within group | 1 | 5 | 0 | 10.25 | 12 | 12.6 | 17 | 19 |
|  |  | 2 | 58 | 0 | 2 | 3 | 6.19 | 12 | 22 |
|  | Between group | 1 & 2 | 63 | 253 | 258 | 259 | 260 | 261 | 268 |
|  | Overall | 1 & 2 | 63 | 0 | 2 | 4 | 43.91 | 14 | 268 |

**Supplementary Table S8:** Statistics of pairwise SNP distances within subtype group

| Category | Subtype | Number of isolates | Pairwise SNP distances | | | | | |
| --- | --- | --- | --- | --- | --- | --- | --- | --- |
|  |  |  | Min | Q1 | Median | Mean | Q3 | Max |
| Before recombination filtering (core genome SNPs) | 2A | 41 | 0 | 1 | 3 | 3.693 | 5 | 15 |
|  | 2B | 9 | 0 | 4.5 | 12.5 | 11.33 | 15 | 28 |
|  | 2C | 4 | 2 | 2 | 2 | 2 | 2 | 2 |
|  | 1D | 2 | 0 | 0 | 0 | 0 | 0 | 0 |

**Supplementary Table S9:** List of major virulence genes identified based on *in silico* genotyping

| Gene | Presentation in isolates (%) | | |
| --- | --- | --- | --- |
|  | Clade 1 | Clade 2 | Overall |
| *esp* | 100.00 | 100.00 | 100.00 |
| *acm* | 100.00 | 100.00 | 100.00 |
| *ebpB* | 80.00 | 0.00 | 6.35 |
| *scm* | 100.00 | 100.00 | 100.00 |
| *sgrA* | 100.00 | 100.00 | 100.00 |
| *efaA* | 100.00 | 100.00 | 100.00 |
| *bopD* | 100.00 | 100.00 | 100.00 |
| *cpsB/cdsA*-EFAU085_01747 | 100.00 | 100.00 | 100.00 |
| *uppS* | 100.00 | 100.00 | 100.00 |
| *pilA* | 80.00 | 31.03 | 34.92 |
| *pilB* | 100.00 | 22.41 | 28.57 |
| *pilE* | 100.00 | 1.72 | 9.52 |
| *pilF* | 100.00 | 1.72 | 9.52 |
| *sagA* | 100.00 | 100.00 | 100.00 |

**Supplementary figures**


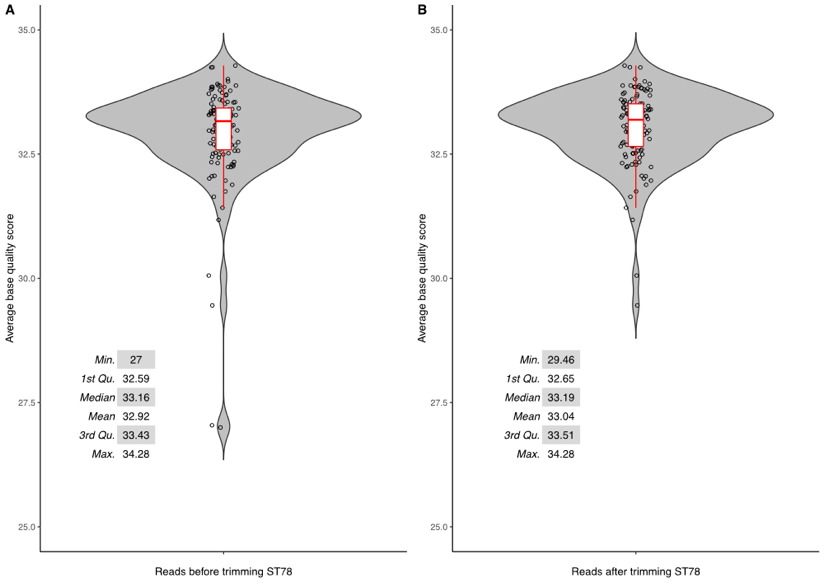


Supplementary Figure S1: Average base quality of the short reads before and after quality control.


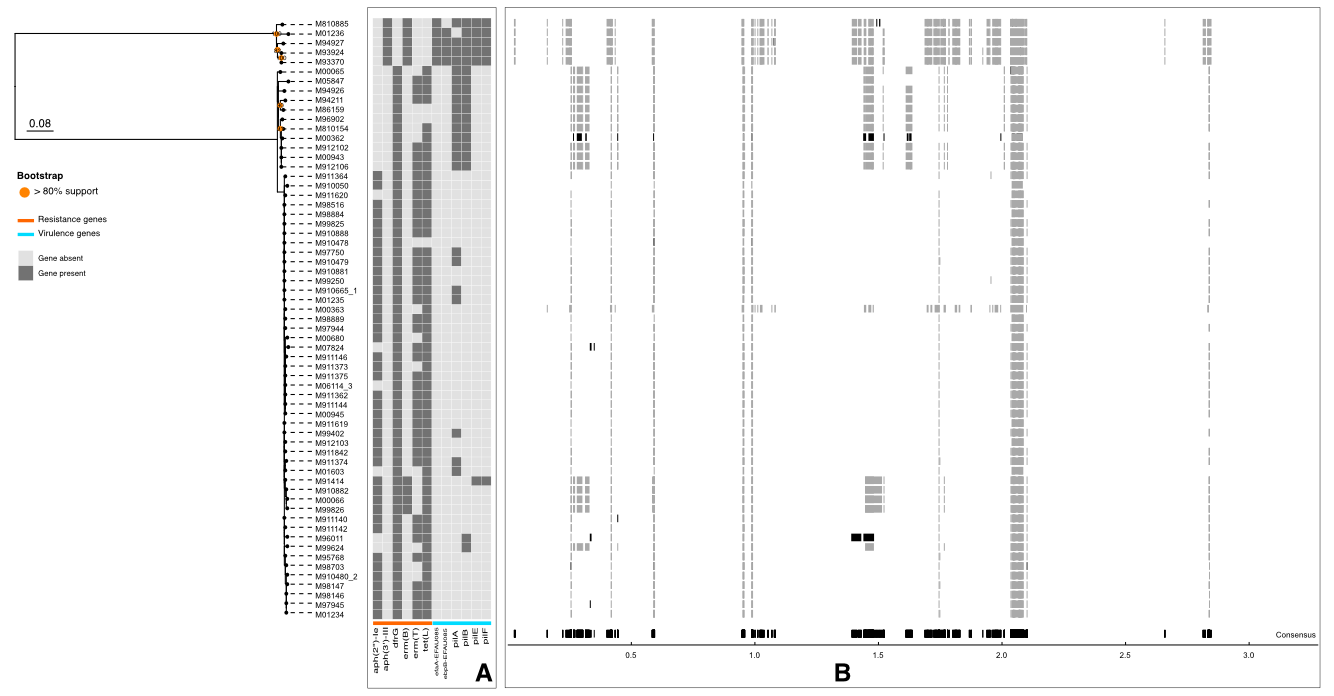


Supplementary Figure S2: Phylogenetic tree and putative recombination region of study isolates VREfm ST78. A. Several distinct resistance and virulence genes. B. Recombination blocks in the pseudo-genome alignment based on core genome SNPs identified by Gubbins. Vertical and horizontal axis represent the order of isolates in the phylogenetic tree and position of nucleotides in the reference genome.


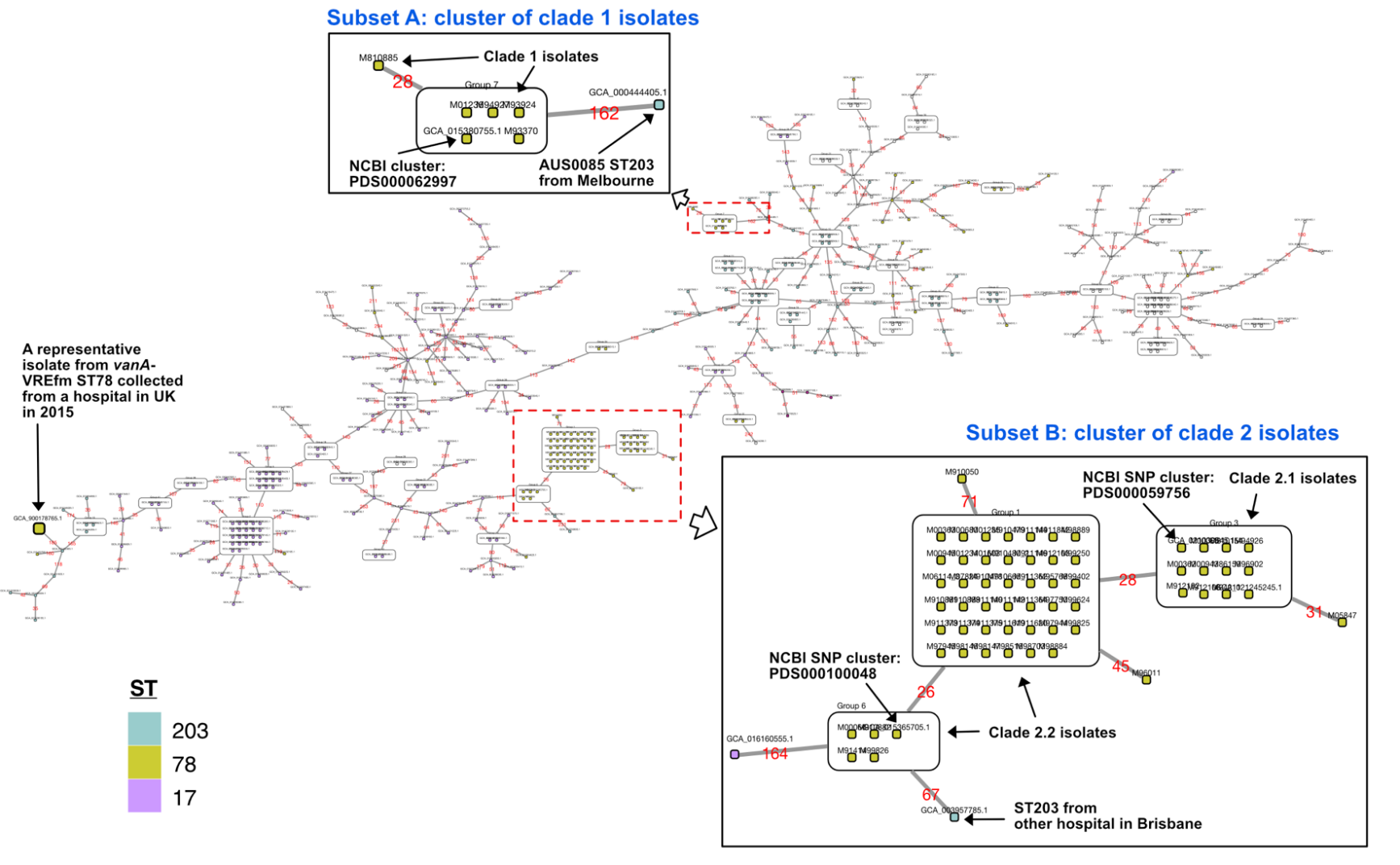


Supplementary Figure S3: Minimum spanning tree of cluster cgMLST distances (cut-off of ≤25 alleles).

The 442 isolates were displayed, consist of ST78 from study isolates, ST78 and its SLVs from NCBI SNP cluster and RefSeq. Node represents isolates. Link denotes pairwise cgMLST distances, with number allele differences were shown in red text. Node colour represents ST. Subset A and B shows where ST78 clade 1 and 2 isolates were clustered, respectively. Isolates of interest, including an ST203 from another hospital in Brisbane and other ST78 isolates from NCBI Pathogen cluster were shown by the arrow.


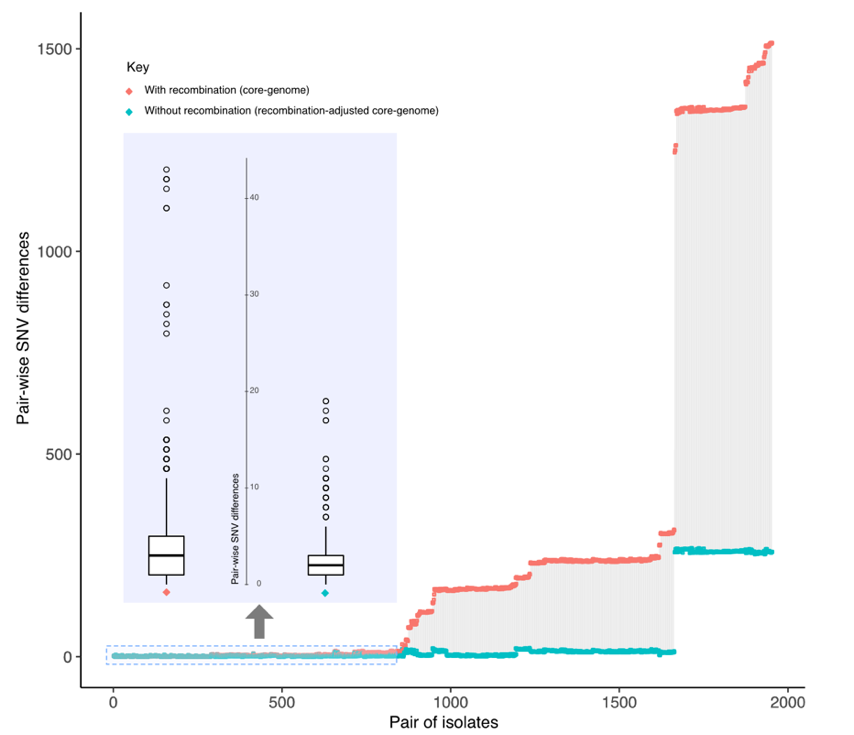


Supplementary Figure S4: Distribution of pairwise SNP distances with and without recombination. Vertical and horizontal axis represents number of pairwise SNP distances and all pair of VREfm ST78 isolates, respectively. Red and teal colour indicate SNP distances of isolates before and after recombination filtering, respectively. Boxplot in purple-shaded area shows the distribution of SNP distances on isolates that have minimal recombination event (0-50 SNPs).


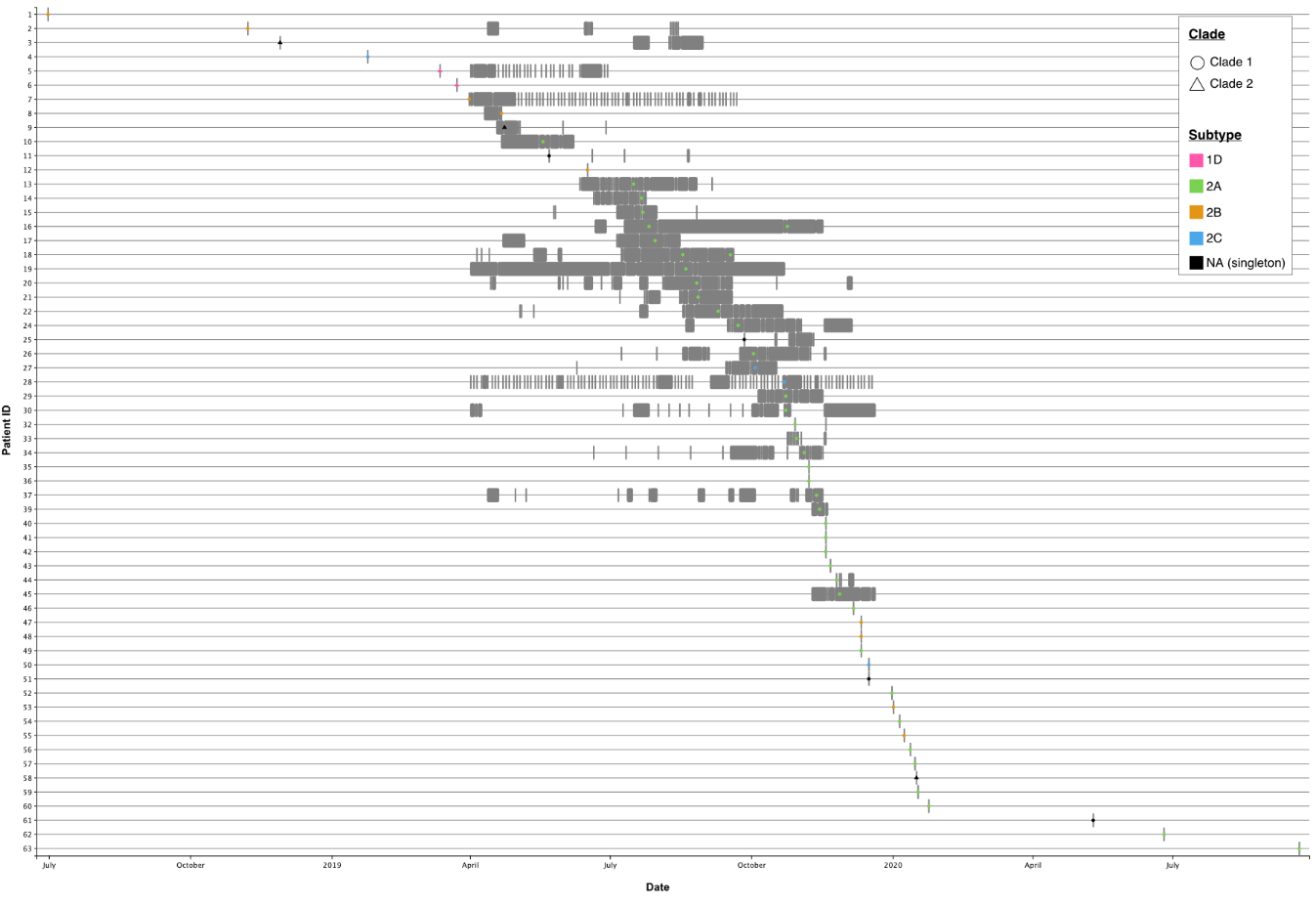


Supplementary Figure S5: Timeline of patient stay.

Horizontal grey line represents patient stay in all wards (not colour coded as too many wards). The length indicates the duration of stay. Spot indicates isolate collection. The spot symbol denotes the clade group and the colour represents subtype.


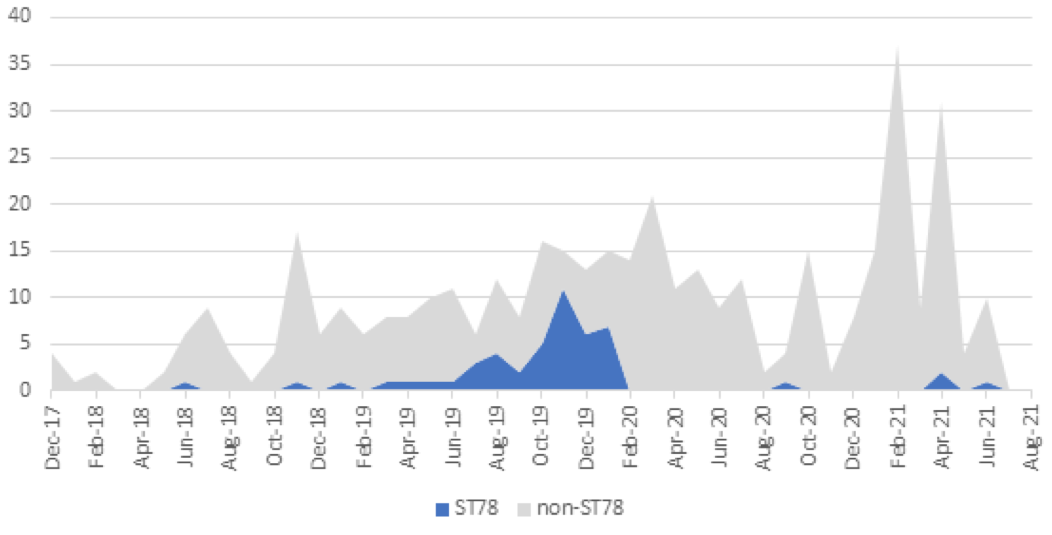


Supplementary Figure S6: Area chart shows the epidemic curve of VREfm ST78 outbreak and other non-ST78 strains in the hospital.


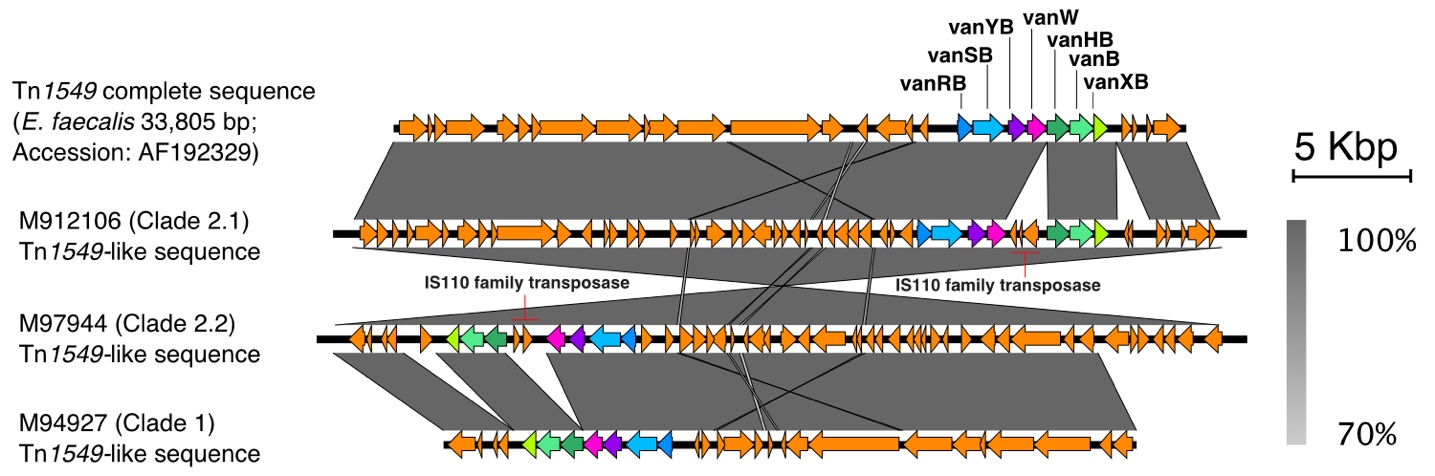


Supplementary Figure S7: Comparison of *Tn1549-*like transposons carrying *vanB* operon in Queensland ST78 isolates. Alignment of *Tn1549* sequence to *Tn1549-*like sequences in Queensland ST78 isolates representing clade 1 (M94927) and clade 2 (M912106 and M97944). The grey line indicates BLASTn hits between pairwise sequences. Arrow indicates coding sequence (CDS). VanB operon was indicated by blue, purple and green colour. IS110 was identified in M912106 and M97944 between *vanW* and *vanHB* genes.


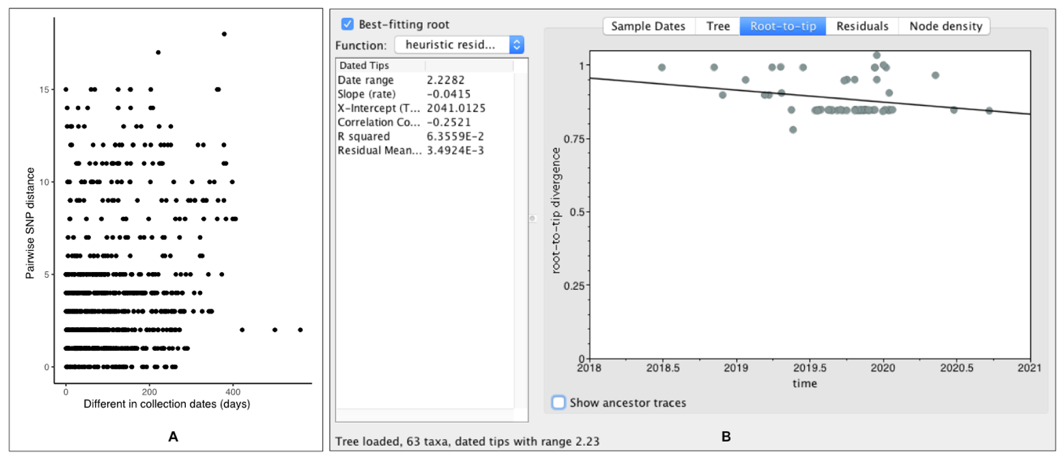


Supplementary Figure S8: Relationship between SNP distance and sample collection date plot.

A. Scatter plot of pairwise SNP distances of Queensland ST78 (subset of distance with maximum of 20 SNPs) against their differences in collection dates (days). B. Temporal analysis of phylogenetic tree (based on core genome SNPs before recombination filtering) against sample collection dates using TempEst v1.5.3.


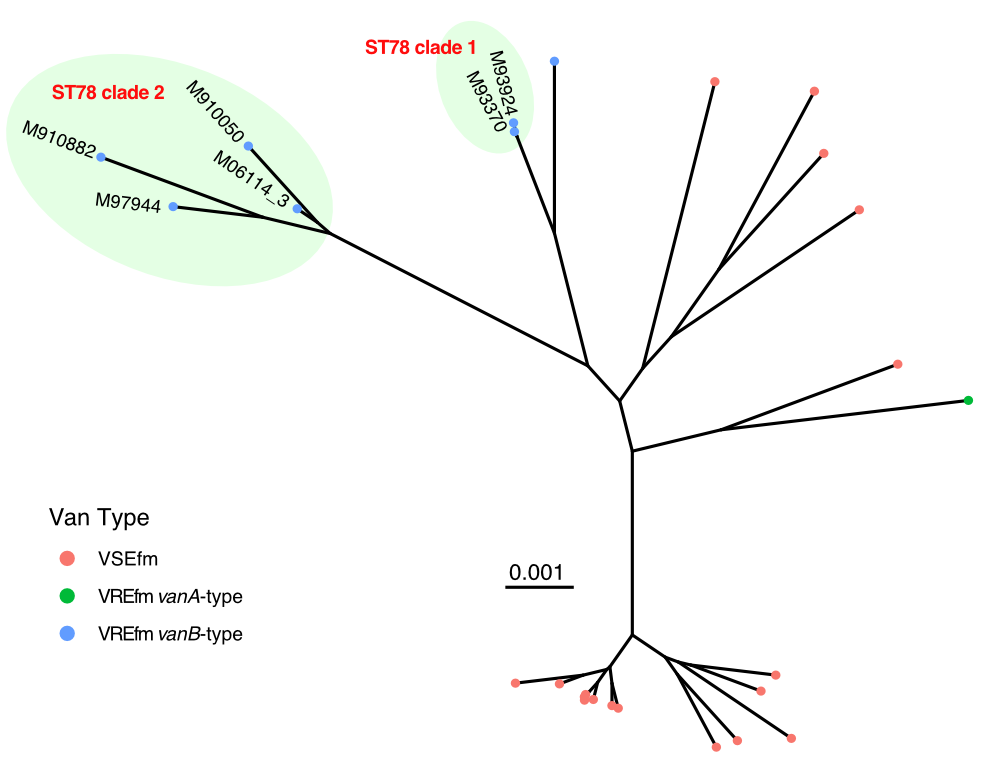


Supplementary Figure S9: Whole genome phylogeny of 26 *E. faecium* isolates.

The whole genome phylogenetic tree built from isolates in the study hospital (6 VREfm ST78, 2 VREfm non-ST78, and 18 VSEfm non-ST78) using Jolytree v1.1b (34). Node colour represents *van* genotype. Node without label represents non-ST78 isolates. Scale denotes pairwise mash distance between genomes.

References

1. Andrews S. FastQC: a quality control tool for high throughput sequence data2010. Available from: <http://www.bioinformatics.babraham.ac.uk/projects/fastqc>.

2. Bolger AM, Lohse M, Usadel B. Trimmomatic: a flexible trimmer for Illumina sequence data. Bioinformatics. 2014;30(15):2114-20.

3. Bankevich A, Nurk S, Antipov D, Gurevich AA, Dvorkin M, Kulikov AS, et al. SPAdes: a new genome assembly algorithm and its applications to single-cell sequencing. J Comput Biol. 2012;19(5):455-77.

4. Gurevich A, Saveliev V, Vyahhi N, Tesler G. QUAST: quality assessment tool for genome assemblies. Bioinformatics. 2013;29(8):1072-5.

5. Inouye M, Dashnow H, Raven LA, Schultz MB, Pope BJ, Tomita T, et al. SRST2: Rapid genomic surveillance for public health and hospital microbiology labs. Genome Med. 2014;6(11):90.

6. Bortolaia V, Kaas RS, Ruppe E, Roberts MC, Schwarz S, Cattoir V, et al. ResFinder 4.0 for predictions of phenotypes from genotypes. J Antimicrob Chemother. 2020;75(12):3491-500.

7. Chen L, Yang J, Yu J, Yao Z, Sun L, Shen Y, et al. VFDB: a reference database for bacterial virulence factors. Nucleic Acids Res. 2005;33(Database issue):D325-8.

8. Li H, Durbin R. Fast and accurate short read alignment with Burrows-Wheeler transform. Bioinformatics (Oxford, England). 2009;25(14):1754-60.

9. Koboldt DC, Zhang QY, Larson DE, Shen D, McLellan MD, Lin L, et al. VarScan 2: Somatic mutation and copy number alteration discovery in cancer by exome sequencing. Genome Res. 2012;22(3):568-76.

10. Katz LS, Griswold T, Williams-Newkirk AJ, Wagner D, Petkau A, Sieffert C, et al. A Comparative Analysis of the Lyve-SET Phylogenomics Pipeline for Genomic Epidemiology of Foodborne Pathogens. Frontiers in Microbiology. 2017;8.

11. Li H. A statistical framework for SNP calling, mutation discovery, association mapping and population genetical parameter estimation from sequencing data. Bioinformatics. 2011;27(21):2987-93.

12. Croucher NJ, Page AJ, Connor TR, Delaney AJ, Keane JA, Bentley SD, et al. Rapid phylogenetic analysis of large samples of recombinant bacterial whole genome sequences using Gubbins. Nucleic Acids Research. 2015;43(3).

13. Page AJ, Taylor B, Delaney AJ, Soares J, Seemann T, Keane JA, et al. SNP-sites: rapid efficient extraction of SNPs from multi-FASTA alignments. Microb Genom. 2016;2(4):e000056.

14. Stamatakis A. RAxML version 8: a tool for phylogenetic analysis and post-analysis of large phylogenies. Bioinformatics. 2014;30(9):1312-3.

15. Permana B, Harris PNA, Roberts WL, Cuddihy T, Paterson D, Forde BM, et al. HAIviz: Healthcare-associated infections visualization tool. Website <https://haiviz.beatsonlab.com/>.

16. Yu GC, Smith DK, Zhu HC, Guan Y, Lam TTY. GGTREE: an R package for visualization and annotation of phylogenetic trees with their covariates and other associated data. Methods Ecol Evol. 2017;8(1):28-36.

17. Team RC. R: A Language and Environment for Statistical Computing. 2020.

18. Eaton K. NCBImeta: efficient and comprehensive metadata retrieval from NCBI databases. Journal of Open Source Software. 2020;5:1990.

19. Treangen TJ, Ondov BD, Koren S, Phillippy AM. The Harvest suite for rapid core-genome alignment and visualization of thousands of intraspecific microbial genomes. Genome Biol. 2014;15(11).

20. Cuddihy T, Harris PNA, Permana B, Beatson SA, Forde BM. CATHAI: cluster analysis tool for healthcare-associated infections. Bioinformatics Advances. 2022;2(1).

21. Gouliouris T, Coll F, Ludden C, Blane B, Raven KE, Naydenova P, et al. Quantifying acquisition and transmission of Enterococcus faecium using genomic surveillance. Nat Microbiol. 2021;6(1):103-+.

22. Campbell F, Didelot X, Fitzjohn R, Ferguson N, Cori A, Jombart T. outbreaker2: a modular platform for outbreak reconstruction. BMC Bioinformatics. 2018;19(Suppl 11):363.

23. Vines MPaNBaKCaK. CODA: convergence diagnosis and output analysis for MCMC. R News. 2006;6:7-11.

24. Willems RJ, Homan W, Top J, van Santen-Verheuvel M, Tribe D, Manzioros X, et al. Variant esp gene as a marker of a distinct genetic lineage of vancomycin-resistant Enterococcus faecium spreading in hospitals. Lancet. 2001;357(9259):853-5.

25. Nallapareddy SR, Weinstock GM, Murray BE. Clinical isolates of Enterococcus faecium exhibit strain-specific collagen binding mediated by Acm, a new member of the MSCRAMM family. Mol Microbiol. 2003;47(6):1733-47.

26. Hendrickx AP, van Luit-Asbroek M, Schapendonk CM, van Wamel WJ, Braat JC, Wijnands LM, et al. SgrA, a nidogen-binding LPXTG surface adhesin implicated in biofilm formation, and EcbA, a collagen binding MSCRAMM, are two novel adhesins of hospital-acquired Enterococcus faecium. Infect Immun. 2009;77(11):5097-106.

27. Sillanpaa J, Nallapareddy SR, Prakash VP, Qin X, Hook M, Weinstock GM, et al. Identification and phenotypic characterization of a second collagen adhesin, Scm, and genome-based identification and analysis of 13 other predicted MSCRAMMs, including four distinct pilus loci, in Enterococcus faecium. Microbiology (Reading). 2008;154(Pt 10):3199-211.

28. Hufnagel M, Koch S, Creti R, Baldassarri L, Huebner J. A putative sugar-binding transcriptional regulator in a novel gene locus in Enterococcus faecalis contributes to production of biofilm and prolonged bacteremia in mice. J Infect Dis. 2004;189(3):420-30.

29. Teng F, Kawalec M, Weinstock GM, Hryniewicz W, Murray BE. An Enterococcus faecium secreted antigen, SagA, exhibits broad-spectrum binding to extracellular matrix proteins and appears essential for E. faecium growth. Infect Immun. 2003;71(9):5033-41.

30. Lam MM, Seemann T, Tobias NJ, Chen H, Haring V, Moore RJ, et al. Comparative analysis of the complete genome of an epidemic hospital sequence type 203 clone of vancomycin-resistant Enterococcus faecium. BMC Genomics. 2013;14:595.

31. Nallapareddy SR, Singh KV, Okhuysen PC, Murray BE. A functional collagen adhesin gene, acm, in clinical isolates of Enterococcus faecium correlates with the recent success of this emerging nosocomial pathogen. Infect Immun. 2008;76(9):4110-9.

32. Sillanpaa J, Prakash VP, Nallapareddy SR, Murray BE. Distribution of genes encoding MSCRAMMs and Pili in clinical and natural populations of Enterococcus faecium. J Clin Microbiol. 2009;47(4):896-901.

33. Hendrickx APA, Bonten MJM, van Luit-Asbroek M, Schapendonk CME, Kragten AHM, Willems RJL. Expression of two distinct types of pili by a hospital-acquired Enterococcus faecium isolate. Microbiology (Reading). 2008;154(Pt 10):3212-23.

34. Criscuolo A. A fast alignment-free bioinformatics procedure to infer accurate distance-based phylogenetic trees from genome assemblies. Research Ideas and Outcomes. 2019;5.
